# Metagenome-assembled genomes of deep-sea sediments: changes in microbial functional potential lag behind redox transitions

**DOI:** 10.1093/ismeco/ycad005

**Published:** 2024-01-10

**Authors:** Clemens Schauberger, Bo Thamdrup, Clarisse Lemonnier, Blandine Trouche, Julie Poulain, Patrick Wincker, Sophie Arnaud-Haond, Ronnie N Glud, Lois Maignien

**Affiliations:** Hadal & Nordcee, Department of Biology, University of Southern Denmark, Campusvej 55, Odense M 5230, Denmark; Hadal & Nordcee, Department of Biology, University of Southern Denmark, Campusvej 55, Odense M 5230, Denmark; Microbiology of Extreme Environments Laboratory, CNRS, IFREMER, Univ Brest, F-29280 Plouzané, France; Microbiology of Extreme Environments Laboratory, CNRS, IFREMER, Univ Brest, F-29280 Plouzané, France; Génomique Métabolique, Genoscope, Institut François Jacob, CEA, CNRS,University of Évry, Université Paris-Saclay, 91057 Evry, France; Génomique Métabolique, Genoscope, Institut François Jacob, CEA, CNRS,University of Évry, Université Paris-Saclay, 91057 Evry, France; MARBEC, CNRS, IRD, Institut Français de Recherche pour L'Exploitation de la Mer, Univ Montpellier, 34200 Sète, France; Hadal & Nordcee, Department of Biology, University of Southern Denmark, Campusvej 55, Odense M 5230, Denmark; Department of Ocean and Environmental Sciences, Tokyo University of Marine Science and Technology, 4-5-7 Konan, Minato-ku, Tokyo 108-8477, Japan; Microbiology of Extreme Environments Laboratory, CNRS, IFREMER, Univ Brest, F-29280 Plouzané, France

**Keywords:** microbial ecology, geomicrobiology, marine sediments, hadal zone, metagenomics, redox gradients, biogeochemistry, CAZymes

## Abstract

Hadal sediments are hotspots of microbial activity in the deep sea and exhibit strong biogeochemical gradients. But although these gradients are widely assumed to exert selective forces on hadal microbial communities, the actual relationship between biogeochemistry, functional traits, and microbial community structure remains poorly understood. We tested whether the biogeochemical conditions in hadal sediments select for microbes based on their genomic capacity for respiration and carbohydrate utilization via a metagenomic analysis of over 153 samples from the Atacama Trench region (max. depth = 8085 m). The obtained 1357 non-redundant microbial genomes were affiliated with about one-third of all known microbial phyla, with more than half belonging to unknown genera. This indicated that the capability to withstand extreme hydrostatic pressure is a phylogenetically widespread trait and that hadal sediments are inhabited by diverse microbial lineages. Although community composition changed gradually over sediment depth, these changes were not driven by selection for respiratory or carbohydrate degradation capability in the oxic and nitrogenous zones, except in the case of anammox bacteria and nitrifying archaea. However, selection based on respiration and carbohydrate degradation capacity did structure the communities of the ferruginous zone, where aerobic and nitrogen respiring microbes declined exponentially (half-life = 125–419 years) and were replaced by subsurface communities. These results highlight a delayed response of microbial community composition to selective pressure imposed by redox zonation and indicated that gradual changes in microbial composition are shaped by the high-resilience and slow growth of microbes in the seafloor.

## Introduction

Marine sediments are among the largest pools of organic matter on the planet [1]. The metabolic processes of ~10^29^ microbial cells living within marine sediments determine whether organic matter gets buried or mineralized in these systems and have an impact on element cycles on a global scale [2–4]. Microbial mineralization processes in sediments make organic matter more recalcitrant and result in a decreasing availability of organic matter with increasing sediment depth [5]. In parallel to this gradient, microbial respiration sequentially depletes available electron acceptors with increasing sediment depth and causes the development of a redox zonation [6, 7]. The resulting energetic gradient from redox zonation and the increasing recalcitrance of organic matter influence mineralization rates and impose selective pressure on microbes [2, 8–10]. Understanding the relationship between microbial communities and such geochemical gradients is crucial for gaining a mechanistic understanding of the role of sediments in the cycling of carbon and other elements.

Reaching depths of 6000–11 000 m below sea level and stretching thousands of kilometers along subduction zones, hadal trenches are the deepest parts of the ocean [11, 12]. Relative to other deep-sea environments, they are characterized by even greater hydrostatic pressure [13] and high depositional variability [14], as frequent seismicity-driven turbidity currents can transfer large amounts of sediment from the trench slopes to their interiors [15, 16]. Also, the topographic shape of trenches in combination with tidal-fluid dynamics leads to the focusing of organic material in the basins of trenches, resulting in elevated respiratory activities compared to adjacent shallower settings [17]. For instance, in the sediments of the Atacama Trench, oxygen is depleted by aerobic respiration within the 2.6–4.1 cm at hadal depths, while in adjacent abyssal settings, oxygen penetrates ~22 cm into the sediment [18]. The oxic zone is succeeded by a zone of nitrate/nitrite and manganese respiration [19], here referred to as the nitrogenous zone, and extends to a depth of ~8 cm into the sediment [19, 20]. After the depletion of nitrate, ferrous iron produced by iron reduction begins to accumulate [19], while a buildup of hydrogen sulfide was despite the activity of sulfate reducers not observed within the upper 40 cm of sediment [20]. In continental sediments where a similar redox zonation is found, sediment mixing by bioturbation blurs relationships between community composition and redox gradients [21]. By contrast, hadal trench sediments are hardly affected by bioturbation due to the lack of burrowing macroinfauna [14, 22], which makes them great model systems to explore the succession of microbial communities and their functions alongside biogeochemical gradients [23].

Despite the high hydrostatic pressure, hadal sediments are inhabited by diverse microbial communities as indicated by 16S rRNA gene sequencing and shotgun metagenomics [24–26]. Hadal microbial communities exhibit high novelty on the species level, yet mostly belong to well-described microbial phyla and classes with the degree of phylogenetic novelty increasing over oceanic depth [27]. However, most data on hadal sediments originated from the Mariana Trench and may not be representative of the entire hadal realm, which exhibits considerable topographic, environmental, and biogeochemical variations [11, 18]. Microbial communities in hadal sediments clearly differed from those in adjacent abyssal environments and showed strong downcore shifts in community composition at the phylum and class levels that are more extensive than those observed in abyssal sediments [24, 28]. Although data on 16S rRNA gene amplicons do not allow the identification of specific microbes that were actively thriving in these systems, estimated absolute abundances derived by normalizing these data with microbial cell counts suggested that the observed community changes in hadal sediments could only be attributed to the active growth and decay of microbes [28, 29].

It is plausible that these community changes are driven by the changes in electron acceptor availability. This hypothesis would predict that the well-defined boundaries of the oxic/nitrogenous/ferruginous zones are accompanied by changes in the respiratory potential of the community. However, gradients in the availability and reactivity of organic matter in parallel to redox zonation could also be a possible explanation [8, 9]. Prediction of functional potential for organic matter degradation from microbial genomes [30–32] is complicated by the complex nature of organic matter in marine sediments with a mixture of different types of molecules such as nucleic acids, lipids, peptides, and carbohydrates [5]. In addition, distinguishing between anabolic and catabolic enzymes is not straightforward. Carbohydrate-active enzymes (CAZymes) are often specific to particular glycosidic bonds within particular substrates [33], and microbes that degrade complex polysaccharides often possess large arsenals of CAZyme genes [34]. As a significant fraction of organic matter in marine sediments are carbohydrates [5], it is feasible that changes in microbial community composition could be driven by the ability of microorganisms to mineralize carbohydrates. However, we currently lack a clear understanding of the CAZyme repertoires of benthic microbes and the degree to which differences in carbohydrate utilization potential may contribute to niche differentiation or otherwise structure benthic microbial communities in hadal sediments.

Here, we explored how the unique conditions in the hadal zone shape benthic microbial community composition in the Atacama Trench. We tested whether selection due to electron acceptor availability is reflected in the respiratory potential of microbes present in the sediments and how important niche differentiation by differential carbohydrate degradation potentials is for community composition. For this, we produced and genomically analyzed a collection of 1357 nonredundant metagenome-assembled genomes (MAGs) from nine sites within the Atacama Trench region.

## Materials and methods

### Sample collection and categorization

We collected sediment samples from the Atacama Trench region with a multicorer during the SO261 expedition on the R/V Sonne. A total of nine stations were targeted, two on the continental shelf at bathyal (S1, 2560 m) and abyssal (S9, 4050 m) depths, one at abyssal depths on the subducting plate (S7, 5500 m), and six hadal sites along a 430 km long transect within the trench axis (7720–8085 m depth). The recovered sediment cores were immediately processed in a 4 °C cold room, using sterilized equipment and with a vertical resolution of 1 cm down to 10 cm and 2.5 cm thereafter. The final slice of each core was discarded to avoid potential contamination. After homogenizing, samples were transferred to 2 ml cryotubes, frozen to −80 °C, and shipped on dry ice to the laboratory. We classified the samples as oxic, nitrogenous, or ferruginous based on *in situ* oxygen data from the same locations [18] and nitrate penetration depths measured from parallel cores [19], as described previously [28].

### DNA extraction, library construction, and sequencing

DNA was extracted from ~0.25 g of sediment, using the DNeasy PowerSoil Pro Kits (Qiagen, Hilden,Germany) according to the manufacturer’s instructions and stored at −80 °C thereafter. The NEBNext Ultra II DNA Library prep kit (New England Biolabs, MA, USA) was used to construct libraries from 10 ng or less DNA from each sample. After DNA quantification and quality control of the library, 10 nM of each library was applied to cluster generation according to the Illumina Cbot User Guide (Part #15006165). Sequencing of libraries was performed according to the Novaseq 6000 System User Guide Part #20023471 (Illumina, San Diego, CA, USA) in paired-end mode (2 × 150 bp). For further details of library preparation and sequencing, see Trouche *et al*. (2023).

### Assembly, binning, and coverage estimation

We quality filtered the demultiplexed reads, using Illumina-Utils python scripts [36] following previous recommendations by Minoche and colleagues [37]. We then processed all samples from each core using a premade Snakemake workflow [38]. This workflow assembled each sample individually with a minimum contig length of 1000 bp using Megahit [37] (v 1.1) and mapped all reads from samples of the same sediment core against each of its assemblies using bowtie2 [39]. Based on these coverage data, we grouped contigs for each sample into bins with concoct [40], metabat2 [41], and maxbin2 [42] and applied dasTool [43] to aggregate the bins and minimize their estimated contamination and maximize their completion. We then renamed all bins from all samples and selected those that had an estimated completeness of >75% and estimated contamination of <25% contamination (CheckM) [44]. We used dREP [45] (0.98 average nucleotide identity cutoff) to dereplicate the MAGs. Completeness and redundancy of the dereplicated MAGs were then estimated again by anvi’o [46] based on single-copy core gene collections [47] and filtered to those with <10% estimated contamination. We then again used a Snakemake workflow [38] to map all reads from all samples against this MAG collection using bowtie2 [39]. We used the fraction of the estimated mean coverage of each MAG from the sum of all mean coverages within a sample as an inference of the approximate relative abundance of each MAG.

### Phylogenetic placements and taxonomic annotations

We used the GTDB-tk classify_wf workflow [48] with the genome taxonomy database (GTDB) database [49] (r207) to assign taxonomy to our MAG collection. We further used the alignment of the 53 and 120 marker genes of archaeal and bacterial MAGs, respectively, generated during the de_novo_wf of GTDB-tk to reconstruct maximum likelihood trees in IQTREE (v2.0.3) [50]. These trees used the LG + F + R7 (*Archaea*) and LG + F + R10 (Bacteria) substitution models [51] and were calculated with 1000 ultrafast bootstrap [52]/SH-aLRT [53] replicates and then visualized using iTol [54]. At the same time, we imported the *de novo* trees from the GTDB-tk workflow into R (v 4.2.0) [55] to quantify the phylogenetic distances within each phylum using the pd command of the picante package [56].

### Functional annotations

We predicted open reading frames of the MAGs using prodigal [57] and annotated predicted genes using KOfamscan [58] and eggNOG-mapper [59] v2.1.2. To estimate respiratory capabilities from the KOfamscan output, we identified MAGs with the (i) aerobic respiration via presence/absence of cytochrome *c*, *cbb3*-type, and *bd* oxidases (≥50% Kyoto Encyclopedia of Genes and Genomes, KEGG, module completeness); (ii) nitrogen respiration via presence/absence of nitrate reductases (napAB, narGHI), nitrite reductases (nrfAH, nirBD, nirK/S), nitric oxide reductases (norBC), nitrous oxide reductases (nosZ); and (iii) sulfur respiration via presence/absence of dissimilatory sulfite reductases (dsrAB). To identify potential iron/manganese reduction capabilities, we searched for presence/absence of homologs of various porin-cytochromes (FeGenie) [60]. The prediction of the eggNOG-mapper was used to determine potential CAZymes [33] in the dataset. These genes were subsequently scanned with SignalP 6.0 [61] to search for signal peptides for excretion.

### Data analysis and visualization

We imported the resulting tables into R (v 4.2.0) for data management, statistical analyses, and visualizations. For this, we used the multiple packages of the tidyverse [62], as well as, ggVennDiagram [63], ggpubr [64], viridis [65], data.table [66], ampvis2 [67], and vegan [68].

## Results

We obtained 153 metagenomes from sediment cores (20–45 cm) collected at nine sites in the Atacama Trench region (see Schauberger *et al*.) [29]. The six hadal sites were located along a 430-km transect along the trench axis and had depths ranging from 7720 to 8085 m, while the abyssal site on the subducting plate, abyssal site on the continental slope, and the one bathyal site were located at 5500, 4050, and 2560 m, respectively (Supplementary Fig. 1). The samples were taken from intact sediment cores, which were sliced at high vertical resolution (see Materials and Methods section). Our dataset consists of over 4 × 10^10^ quality-filtered paired-end reads, with a median of 130 million read pairs per sample (Supplementary Fig. 2). Due to the size of the dataset, we processed the data from each site individually and binned a total of 11 346 MAGs before dereplicating the entire MAG collection with a 98% average nucleotide identity cutoff. The final set of 1357 nonredundant MAGs used for further analysis had a median estimated completeness of 90% (ranging between 75%–100%) and a median estimated redundancy (contamination) of 3% (0%–9.9%, Supplementary Fig. 1).

### Hadal sediments are inhabited by diverse and taxonomically novel microbial communities

**Figure 1:**
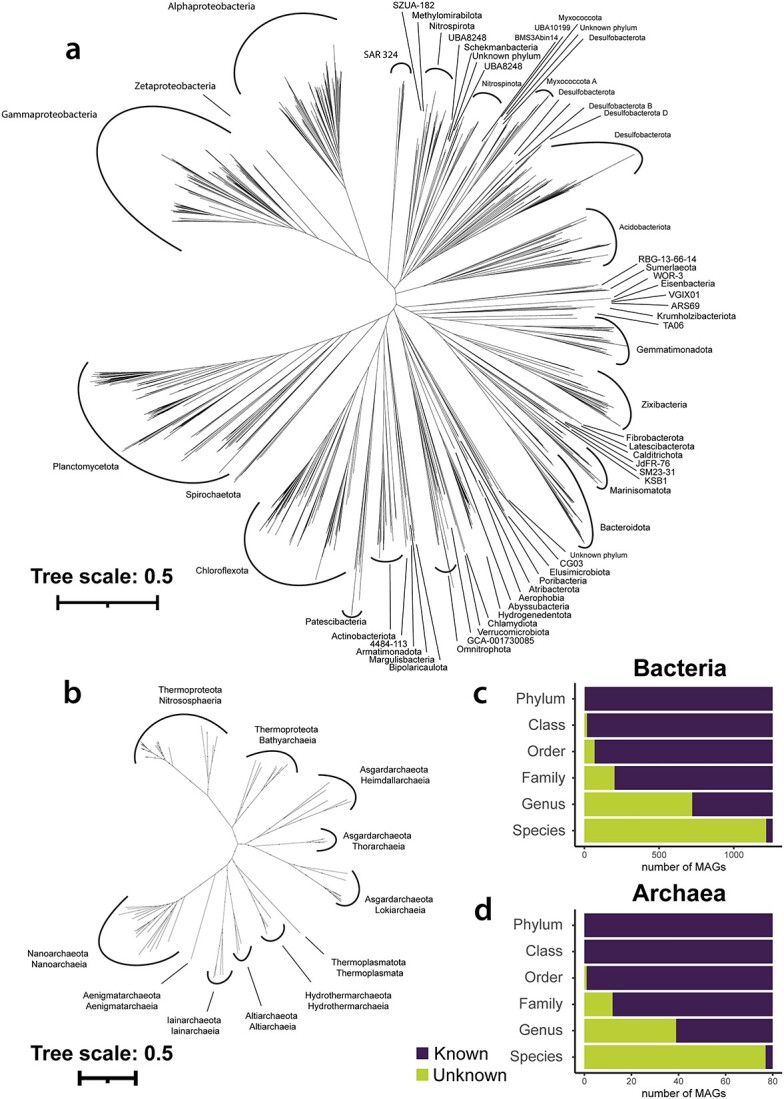
Phylogenomic trees of bacterial (A) and archaeal (B) MAGs, based on the protein alignment of 120 and 53 marker genes, respectively; trees were reconstructed based on maximum likelihood using IQTree and LG + F + R10 (bacteria) and LG + F + R7 (*archaea*) substitution models; labels show bacterial phyla (*Proteobacteria*: class) classifications (A), and archaeal phyla and class classifications; (B) stacked bar plots of classified versus unclassified bacterial (C) and archaeal (D) MAGs (*x*-axis) on different taxonomic levels (*y*-axis), based upon GTDB-TK and GTDB version 207.

We evaluated the taxonomic diversity of the 1357 nonredundant MAGs using the GTDB toolkit [48]. The bacterial domain encompassed 1272 MAGs that spanned over 55 different bacterial phyla, while 85 MAGs were classified into 8 archaeal phyla (Fig. 1). At low taxonomic levels, most MAGs did not get classified by the GTDB toolkit, leaving 97% of all MAGs without species classification and 57% without genus classification. However, at the family level, most MAGs could be classified into known microbial lineages (16% unknown). As six bacterial MAGs could not be classified at the phylum level, we determined the phylogenetic affiliation of all MAGs by using the alignment of the marker genes from the GTDB toolkit and reconstructing phylogenetic trees for both bacteria and archaea (IQTree). Within the bacterial tree, three of these six previously unclassified MAGs (#10039, #10401, #10878) were phylogenetically located within the phylum of UBA8248, which is a close relative to *Schekmanbacteria*. Two MAGs (#4666, #4362) were placed next to Phylum CG03 and thereby close relatives of *Elusimicrobiota*, and #8291 was placed next to a MAG classified as a *Desulfobacterota* (polyphyletic in this tree) between *Myxococcota*, and *Myxococcota_A* (Fig.1A).

To determine how much phylogenetic novelty our MAGs add to the phylogenetic trees of the GTDB, we combined our 1357 nonredundant MAGs with those of the reference collection r207 and reconstructed phylogenetic trees (GTDB toolkit de_novo_wf). With this, we extended the GTDB tree branch lengths by 2.2% in the bacterial domain and 1.8% in the archaeal domain (Supplementary Tables 1 and 2), revealing previously unknown parts of the tree of life. Branch lengths increased by >20% for 14 bacterial phyla and by >50% for six of these phyla. The greatest increases in the bacterial diversity were within relatively unexplored microbial lineages such as *Nitrospinota*, *Schekmanbacteria*, *Zixibacteria*. *Krumholzibacteria*, *Hydrogenedentota*, *Abyssubacteria*, and SAR324. The archaeal tree also expanded significantly, with six MAGs adding >20% branch length to *Hydrothermarchaeota*. Despite being the most diverse groups with highest number of taxa in our dataset, *Chloroflexota*, *Gammaproteobacteria*, and *Alphaproteobacteria* showed only modest increases in branch length of 6%, 2%, and 2%, respectively. Hence, although our study revealed significant phylogenetic diversity within less explored microbial groups, most MAGs showed a high phylogenetic similarity to the reference collection.

### Hadal microbial communities are ecologically and genomically dissimilar to those of abyssal and bathyal settings

To determine the relative abundances of MAGs, we conducted a read mapping analysis, aligning all 153 metagenomes against our dereplicated MAG collection. The MAG collection recruited an average of ~36 ± 0.6% (mean ± standard error) of the obtained reads from the hadal samples (Supplementary Fig. 2). The 24 samples from the hadal nitrogenous zone recruited the highest proportion of reads (42 ± 1%), followed by the 66 samples from the ferruginous zone (36 ± 0.4%) and the 20 samples from the oxic zone (30 ± 1.4%). Read recruitment was lower in samples from the abyssal (28 ± 1% at S7 and 21 ± 0.8% at S9) and bathyal (14 ± 0.7% at S1) sites. Additionally, we observed higher densities of single nucleotide variants (SNVs) per mapped read in abyssal and bathyal sediments compared to hadal samples (Supplementary Fig. 2). Based upon these mapping results, we then estimated relative abundances within our MAG collection by dividing the mean coverage of each MAG by the sum of mean coverages of all MAGs in each sample. Principal coordinate analysis of Bray–Curtis dissimilarities of relative MAG abundances showed that microbial community compositions in hadal sediments are clearly distinct to those of abyssal and bathyal settings, even when only comparing samples from the same redox zones (Supplementary Fig. 3). Hence, the hadal community is dissimilar ecologically (relative abundances) and genomically (SNVs) to those of abyssal and bathyal sediments even under similar biogeochemical conditions.

### Microbial communities in hadal sediments change gradually over sediment depth, with the ferruginous zones emerging as a hotspot of microbial diversity

**Figure 2:**
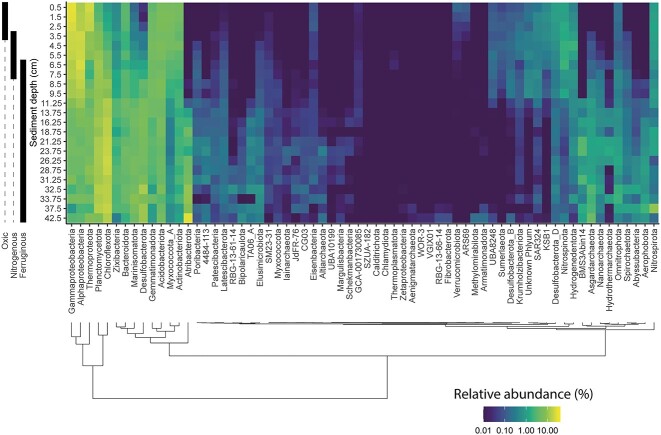
Heatmap of mean relative abundances of microbial phyla and proteobacterial classes (*x*-axis) across six hadal sites plotted against sediment depth (*y*-axis) across six hadal sites; relative abundances of individual MAGs were estimated by dividing the mean coverage of each MAG by the sum of mean coverage of all MAGs within a given sample; the *x*-axis was ordered by hierarchical clustering using Ward’s criterion on Euclidean distances of median abundances.

The changes in community composition were accompanied by high-level taxonomic changes (phyla and classes) with sediment depth (Fig. 2, Supplementary Fig. 3). MAGs belonging to *Gammaproteobacteria* (28 ± 1.0%, mean relative abundance ± standard error), *Alphaproteobacteria* (16 ± 1.0%), and *Thermoproteota* (15 ± 1.4%) dominated the oxic zone, whereas those MAGs associated with *Thermoproteota* to a large extent consisted of putative ammonia oxidizing *Nitrososphaeria*. From the surface to the nitrogenous zone, the relative abundance of *Gammaproteobacteria* decreased to 19 ± 1.5% (Welch’s *t*-test, *p*  <  .05), while the relative abundance of *Alphaproteobacteria* increased to 23 ± 1.6% (Welch’s *t*-test, *p*  <  .05). *Thermoproteota* decreased in abundance to ~5% (Welch’s *t*-test, *p*  <  .05) in the nitrogenous zone, while anaerobic ammonia-oxidizing *Brocadiae* (*Planctomycetes*) showed here its highest relative abundances of ~5 ± 0.9% (Welch’s *t*-test, *p* <  .05) before disappearing again in the ferruginous zone. *Chloroflexota* were the most abundant phylum in the ferruginous zone with only 16 ± 0.6% relative abundance, while both *Alpha*- (9 ± 0.9%) and *Gammaproteobacteria* (7 ± 0.6%) remained moderately abundant. This resulted in more evenly distributed rank abundance curves in the ferruginous zone relative to the oxic and nitrogenous zones, which were rather dominated by a few abundant microbial lineages. The ferruginous zone harbors low-abundance groups, many of which were barely present or undetectable at the sediment surface but increased in relative abundance either gradually over sediment depth or only after the beginning of either the nitrogenous or ferruginous zones. Hence, the ferruginous zone appears to offer niches for a wide range of diverse microbes rather than selecting for few fast-growing lineages.

### Community change within the oxic zone and between oxic and nitrogenous zones is not due to differences in terminal oxidases

The composition of microbial communities in hadal sediments seems to be closely linked to the redox gradient and the accessibility of electron acceptors [15]. We evaluated the correlation between electron acceptor availability and the predicted respiratory capability of MAGs along the redox gradient in hadal sediments based on the presence or absence of genes for key enzymes involved in respiration and metal reduction. We focus on mean abundances of MAGs with different predicted respiratory capabilities within each sediment horizon across the six hadal sites, while acknowledging potential biases from shifting redox conditions (± 2 cm).

**Figure 3:**
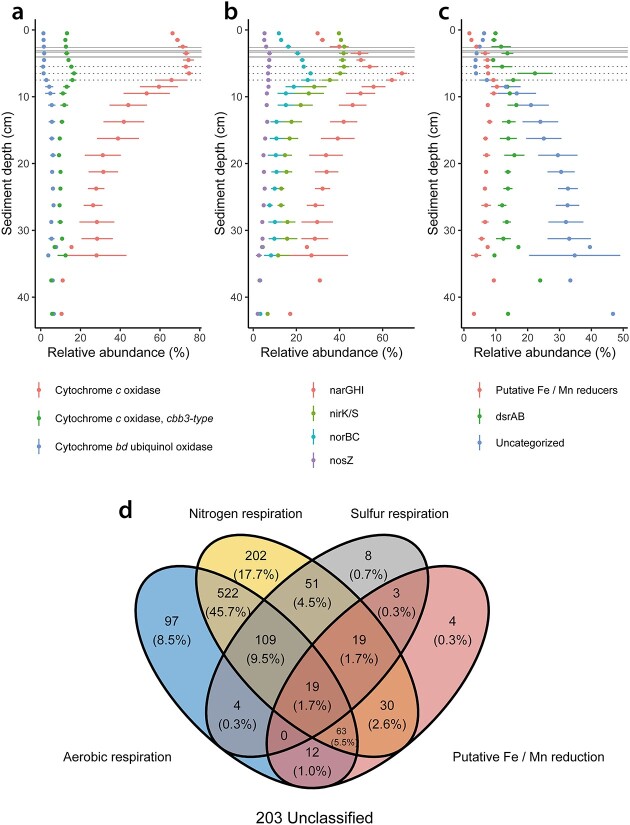
Mean of relative abundances and standard error of MAGs with a selection of terminal oxidases (A), nitrogen respiratory enzymes (B) and genes for sulfur respiration, iron/manganese respiration, as well as MAGs without any respiratory genes (C) across six hadal sites; continuous and dotted horizontal lines represent oxygen and nitrate penetration depths, respectively; (D) Venn diagram illustrating estimated capabilities for aerobic, nitrogen, sulfur, and iron/manganese respiration of MAGs and depicts the overlaps between the four groups, highlighting the number of genomes capable of performing multiple respiratory processes.

There was no clear indication that the presence or concentration of oxygen controlled the relative abundances of MAGs with genes for cytochrome *c* oxidases, *cbb3*-type cytochrome *c* oxidases, and cytochrome *bd* ubiquinol oxidases (≥ 50% module completeness, Fig. 3A, Supplementary Fig. 4). At the hadal sediment surface, MAGs with cytochrome *c* oxidases encompassed a relative abundance of 66 ± 0.7% (mean ± standard error), those with *cbb3*-type cytochrome *c* oxidases 13 ± 0.4%, and those with cytochrome *bd* ubiquinol oxidases only 1.3 ± 0.1%. The Complex IV of chemolithoautotrophic ammonia-oxidizing lineage *Nitrosopumilaceae* is dissimilar [69] to those of bacteria and not covered with our 50% KEGG module completeness cutoff. Considering that *Nitrosopumilaceae* account for ~20 ± 1.0% of the relative abundance of the microbial community at the sediment surface and adding that to the relative abundance of microbes with cytochrome *c* (including *cbb3-type*) and *bd* ubiquinol oxidases, we estimate that ~86 ± 0.4% of all microbes at the sediment surface were capable of aerobic respiration. *Nitrosopumilaceae* and *Gammaproteobacteria* had peak relative abundances at the sediment surface and declined gradually with sediment depth. However, the overall relative abundance of MAGs with cytochrome *c* oxidases increased with increasing depth and peaked at ~75 ± 1.9% in the middle of the nitrogenous zone, before decreasing exponentially in the ferruginous zone. This is likely because ~63% of MAGs with cytochrome *c* oxidases also have the capability for nitrogen respiration (Fig. 3D). MAGs that exclusively contained terminal oxidases for aerobic respiration exhibited similar relative abundance in the oxic and nitrogenous zones (23 ± 0.8–25 ± 1.6%, Welch’s *t*-test, *p*  =  .2) compared to 11 ± 1.0% in the ferruginous zone. Microbes with annotated cytochrome *bd* ubiquinol oxidases increased in relative abundance once nitrate was depleted, mainly due to the increasing relative abundance of *Bacteroidetes*. We did not observe any relationship between the relative abundance of *cbb3*-type cytochrome *c* oxidases—known for their high oxygen affinity [70]—and oxygen concentrations. MAGs with *cbb3*-type cytochrome *c* oxidases were more abundant in the nitrogenous zone (Welch’s *t*-test, *p*  <  .05) than in the oxic and ferruginous zones, partially due to increases in the chemolithoautotrophic, anaerobic ammonium-oxidizing (anammox) lineage of *Brocadiae Planctomycetes*. Thus, the changes in the microbial community composition occurring from the oxic to the nitrogenous zone were not coupled to electron acceptor availability for most lineages, except for chemolithotrophs such as ammonia oxidizing archaea and anammox bacteria.

### Aerobic and nitrogen respiratory capabilities slowly decline in the ferruginous zone with half-life times of centuries

The relative abundance of MAGs capable of nitrate reduction (≥ 2 genes of narGHI) peaked in the nitrogenous zone at a relative abundance of 38 ± 1.6%, as did MAGs capable of reducing nitric oxide (≥1 gene of norBC) at 15 ± 0.9% (Fig. 3B). This was mainly driven by the rise in relative abundance of *Alphaproteobacteria* and *Planctomycetes* (*Brocadia*) in the nitrogenous zone. Potential nitrite reducers (≥1 gene of nirK/S) decreased from the oxic to the nitrogenous zones from ~32 ± 0.6% to 27 ± 0.9% (Welch’s *t*-test, *p* <  .05), while only a small number of microbes (72 MAGs) seemed capable to reduce nitrous oxide (nosZ) with peaks in relative abundance in the nitrogenous zone at around 6 ± 0.9%. Only five MAGs showed the capability to perform full denitrification.

Potential aerobic and nitrogen respiring MAGs declined exponentially in relative abundance in the ferruginous zone. MAGs with cytochrome *c* oxidases declined from 75 ± 1.9% to 31 ± 9% relative abundance between 6 and 20 cm below the sea floor, while those with narGHI declined here from 38 ± 1.6% to 17 ± 4.6%. To estimate the half-lifetime of cells (represented by MAGs) containing cytochrome *c* oxidases and narGHI, we fitted exponential functions into the exponential decline of relative abundance of these MAGs over sediment depth. This estimation assumed constant total cell abundances across sediment depth [29] and relied on sediment ages derived from lead-210-based annual sedimentation rates within the Trench sites, which varied from 0.29 mm/year to 0.76 mm/year [14]. The estimated half-life times of the aerobic community ranged between 135 and 367 years, with similar estimates for cells with narGHI at 154–419 years (Supplementary Fig. 5). Thus, the change in electron acceptor availability between nitrogenous and ferruginous zones imposed a selective effect on the microbial community and led to gradual and steady changes over sediment depth.

### Potential fermenters replace the aerobic and nitrogen respiring community in the ferruginous zone

We estimated the potential for iron/manganese reduction by the presence/absence of homologs of various porin-cytochromes (FeGenie [60]). Most iron-reducing MAGs belonged to *Zixibacteria* (26), *Planctomycetota* (21), *Gammaproteobacteria* (20), *Desulfobacterota* (18), and *Hydrogenedentota* (15). Only 1.7 ± 0.1% of microbes on the sediment surface were capable of metal reduction, but this increased toward the nitrogenous zone to ~8 ± 0.8% (Welch’s *t*-test, *p* <  .05) mainly due to increases in some *Woeseiales* (*Gammaproteobacteria*), *Hydrogenedentota*, and *Marinisomatota*. These lineages disappeared toward the ferruginous zone and were replaced with *Zixibacteria*, *Abyssubacteria*, and *Desulfobacterota*. Similarly, the relative abundance of microbes with dsrAB peaked in the nitrogenous zone due to *Alphaproteobacteria*. Although this enzyme could be used in the reverse direction to oxidize sulfur [71], the lack of free H_2_S in these sediments (Supplementary Table 3) suggested that these genes might not explain the high relative abundance of these microorganisms. Sulfate reduction in the Atacama trench sediments was not detectable in the nitrogenous zone (Supplementary Table 3). However, the relative abundance of *Desulfobacterota* increased from <1 ± 0.4% in the nitrogenous zone to 13% in the ferruginous zone (Welch’s *t*-test, *p* <  .05). Similarly, MAGs without any of the here analyzed enzymes for respiration increased exponentially in relative abundance to ~33 ± 6.7% in the ferruginous zone, replacing the aerobic and nitrogen respiring microbes. This was mainly driven by *Chloroflexota*, *Atribacterota*, *Acidobacteriota*, and *Planctomycetes*. We consider these MAGs with unclassified respiratory pathways here as putative fermenters, yet they might have the capability to use alternative respiratory pathways. It is also possible that even MAGs with identified respiratory genes may switch to a fermentative lifestyle when their favored electron acceptor is depleted. Within the exponential growth phase of putative fermenters between 6.5 and 9.5 cm, they double every 1.4 cm (18–48 years) and dominate the ferruginous zone in relative abundance, indicating that these microbes are highly competitive once nitrate is gone.

### Relative CAZyme abundances only change alongside redox gradients

Most microbes in marine sediments are heterotrophs and a significant fraction of organic material in marine sediments is carbohydrate [2]. We therefore assessed whether changes in microbial community composition between redox zones—particularly oxic and nitrogenous zones—can be explained by differences in microbes’ genomic capacities for carbohydrate degradation [12, 23]. We assessed the distribution of CAZymes genes in our collection of MAGs using sequence-based classification of glycoside hydrolases (GHs) and polysaccharide lyases (PLs). Multiple GH and PL, in conjunction with carbohydrate-binding modules, are frequently combined to larger unified enzyme structures [72]. A potential caveat is the lack of differentiation between putative anabolic and catabolic CAZymes, given the relatively unexplored utilization of these enzymes by environmental microbes [73]. Because large, complex carbohydrates must be degraded extracellularly, we therefore screened the identified CAZyme genes for signal peptides indicative of secretion pathways. Approximately 46% of the enzymes encoded by 12 358 CAZyme genes were predicted to have the potential to be excreted, primarily through the Sec signal pathway (Supplementary Fig. 6).

**Figure 4:**
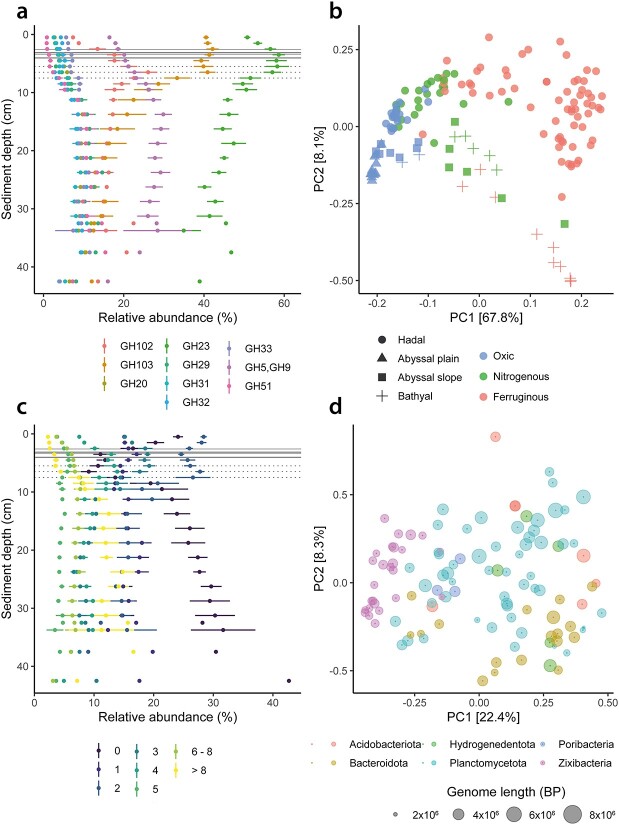
(A) Relationship between the abundance of CAZymes and sediment depth; the plot displays the mean and standard error of the sum of relative abundances of the 10 most abundant CAZymes within microbial genomes against sediment depth; continuous and dotted horizontal lines represent oxygen and nitrate penetration depths, respectively; (B) PCA plot of relative CAZyme abundances across all samples; the colors indicate redox zones, while the different symbol shapes represent the sampling locations; (C) relationship between number of CAZymes within microbial genomes and sediment depth; the plot displays the mean and standard error of the sum of relative abundances of microbial genomes that possess different numbers of CAZyme genes, ranging from zero to more than eight, against sediment depth; continuous and dotted horizontal lines represent oxygen and nitrate penetration depths, respectively; (D) PCA plot of relative CAZyme composition within microbial genomes that possess more than eight different CAZyme families; color indicates phylum classifications of genomes, while the dot-sizes reflects the size of microbial genomes.

We then surveyed the distribution of different CAZyme genes by considering, for each CAZyme gene, the total relative abundance of all MAGs containing that CAZyme gene. MAGs containing the CAZyme genes of peptidoglycan lyases GH23, GH103, and GH102, which have been demonstrated to be critical in anabolic cell-wall synthesis for certain microbes [74], were highest in relative abundance (Fig. 4A). The CAZyme gene abundance of GH23 and GH102 peaked in the nitrogenous zone before steeply declining in the ferruginous zone, as did MAGs containing GH103. The changes in CAZyme gene abundance of GH23 and GH103 were mainly controlled by the abundance of *Alpha*- and *Gammaproteobacteria*, while the relative abundance of GH102-containing MAGs was mainly influenced by *Planctomycetes* and *Alphaproteobacteria*. These three ubiquitous families of peptidoglycan lyases were often the only potentially extracellular CAZymes present in Alpha- and Gammaproteobacterial MAGs in our dataset, indicating that niche differentiation between Alpha- and Gammaproteobacterial MAGs was unlikely driven by their potential for extracellular carbohydrate degradation. Furthermore, this shows that the CAZyme gene downcore trends may have been driven to a large extent by factors other than those associated with carbohydrate degradation. Still, out of 108 different CAZyme genes found in this study, 78 peaked in relative abundance in the ferruginous zone, many of which are more certainly associated with catabolic processes [75, 76]. Thus, the ferruginous zone was enriched in MAGs which encoded potentially extracellular CAZymes.

To determine if the relative CAZyme gene abundances and their trends over sediment depth (Fig. 4, a) were unique to hadal sediments, we performed a principal component analysis (PCA) on Hellinger-transformed CAZyme abundances (Fig. 4B). Although there was some separation between hadal, abyssal, and bathyal samples, most of the dissimilarity between samples was mainly associated with redox zonation. Changes in relative CAZyme gene abundances only occurred when redox conditions changed. This was not linked to organic carbon concentrations, as in fully oxic abyssal plain sediments [18], CAZyme gene abundances were constant even though organic carbon concentrations decreased strongly with sediment depth [29] (Supplementary Fig. 7).

### MAGs with a high number of excreted CAZymes increase toward the ferruginous zone

The degradation of complex carbohydrate molecules usually requires multiple CAZymes and even minor variations in the number of CAZymes within a family can produce substantial differences in carbohydrate degradation potential [26]. Thus, the number of CAZyme genes in a MAG can be seen as an indicator of its capacity for complex carbon degradation [77]. We divided the MAGs into eight groups based on their number of CAZymes with signal peptides for excretion (Supplementary Fig. 8). This revealed three notable trends: (i) MAGs with two excreted CAZymes were the most abundant group on the surface of hadal sediments (Fig. 4C) but decreased with depth due to the decreasing abundance of *Alpha*- and *Gammaproteobacteria*. (ii) MAGs without any excreted CAZymes decreased toward the nitrogenous zones at all sites, driven by changes in abundance of *Thermoproteota* (*Nitrosopumilaceae*), before becoming the most abundant group in the ferruginous zone due to *Chloroflexota*. (iii) MAGs with more than eight excreted CAZymes steadily increased in relative abundance with sediment depth at all hadal sites, as well as the abyssal slope and bathyal site, but not in the fully oxic abyssal plain site (Supplementary Fig. 9). These MAGs with high numbers of distinct CAZymes were significantly more abundant (Welch’s *t*-test, *p*  <  .05,) in the hadal ferruginous zone (12 ± 0.5%) than in the bathyal ferruginous zone (8%) and often belonged to deep-biosphere taxa within *Planctomycetota*, *Zixibacteria*, and *Hydrogenedentota* [10, 21, 78].

### Phylum-level dissimilarities in CAZyme contents of MAGs that possess more than eight excreted CAZymes

To determine potential niche differentiations between complex carbohydrate degrading microbial phyla, we tested if the CAZyme composition of MAGs was phylogenetically conserved via PCA of the Hellinger-transformed CAZyme gene counts of MAGs with over eight excreted CAZymes in their genomes (Fig. 4D, Supplementary Fig. 10). *Planctomycetota*, *Zixibacteria*, and *Bacteroidota* were the most prevalent phyla in this analysis, with 53, 26, and 19 MAGs, respectively, and their CAZyme compositions were significantly dissimilar from each other despite large overlaps between *Planctomycetota* and *Bacteroidota* (pairwise Permutational analysis of variance, PERMANOVA, *p*  <  .05, p.adjust = “fdr”). The CAZyme composition of *Zixibacteria* was distinct from other microbial lineages, mainly because they had many GH6 (cellulase) genes, while dissimilarities between *Bacteroidota* and *Planctomycetota* were mainly due to differences in their copy numbers of multiple GH families. Also, *Poribacteria* differed from other microbial lineages as it was enriched in GH15 (glucoamylase) genes. Hence, our data indicated that, among lineages that degrade complex carbohydrates, CAZyme contents are phylogenetically conserved.

## Discussion

Our results indicate that despite the high hydrostatic pressures in the hadal realm, benthic microbial communities in hadal sediments are diverse and span about a third of all known microbial phyla. Widespread microbial lineages [8, 79, 80] such as *Alphaproteobacteria*, *Gammaproteobacteria*, and *Chloroflexota*, were the most abundant and most numerous MAGs in our dataset. MAGs belonging to these lineages were also the least phylogenetically novel, indicating that they are closely related to organisms that have already been sequenced and added to the GTDB [49]. In turn, rarer lineages found in deeper sediment layers were most phylogenetically novel. Although subsurface sediments are probably less well-covered in public databases than surficial sediments, it is also possible that these lineages were only detectable in our samples because of the unique biogeochemical conditions in hadal sediments. Minimal bioturbation [14], centimeter-scale redox zones [18, 35], and substantial organic carbon content in anoxic layers [81] that are not sulfidic (Supplementary Table 3) set hadal sediments apart from other marine sediments.

Still, ecological dissimilarity scaled with oceanic depth in this dataset, a finding in agreement with previous 16S rRNA gene amplicon studies [10, 11, 15], as well as with the density of single nucleotide variations (SNVs) per mapped read. Although the higher SNV densities in abyssal and bathyal MAGs may be artifacts of read mapping, the systematic increase in SNV density with decreasing water depth suggests a selective effect of ocean depth resulting in significant strain-level variations between hadal microbes and their relatives from shallower environments.

We approximated the respiratory capabilities of the MAGs based on sequence-based annotation of their terminal oxidase genes as well as genes involved in terminal electron accepting processes in anaerobic respiration. However, microbial respiration is a complex process [82], and MAGs are incomplete representations of microbial genomes [83]. Furthermore, genes can be mis-annotated [59], respiratory enzymes can serve multiple non-respiratory functions [84], and microbes may switch to fermentative lifestyles once their electron acceptors become depleted [85]. Yet, although our estimates should not be seen as precise predictions of the respiratory activity or capability of any specific MAG, the high-level trends over the entire collection of 1357 MAGs alongside the redox gradient should account for these uncertainties.

Our data suggested that the gradual community change between the oxic and nitrogenous zones was unlikely to be driven by respiratory capacities, except for nitrifiers and anammox bacteria, as most aerobic microbes were capable of nitrogen respiration. Aside from potential biogeochemical reasons, e.g. the onset of manganese reduction in the nitrogenous zone [35], microbe-microbe interactions between chemolithoautotrophs and symbionts [86] could also play a role for the community changes between oxic and nitrogenous zones.

The strongest trend was the exponential decay of aerobic and nitrogen-respiring microbes in the ferruginous zone with estimated half-lives of centuries, and the parallel growth of putative fermenters with estimated doubling times of several decades. Interestingly, these gradual changes in microbial community composition occurred without drastic changes in microbial cell abundances [29] despite the stark energetic differences between different respiratory pathways (e.g. aerobic respiration vs sulfate reduction). Additionally, once the gradual community shift had mostly ended within the ferruginous zone, the microbial community did not grow to abundances greater than ~5 × 10^7^ cells/ml [29] despite stable redox conditions and substantial amounts of organic carbon [29]. Overall, it appears that the abrupt transition from the nitrogenous and to the ferruginous zone produces a gradual change in community composition and associated functions because microbes in hadal sediments seem to grow slowly and seem to be highly resistant to decay, resulting in a delayed response of the community to shifting selective pressures. Delayed responses in microbial community composition to changing redox conditions were previously observed also in non-hadal sediments, as well as in redox changes beyond those of oxic–nitrogenous–ferruginous zones (e.g. sulfidic—methanic) [8, 87, 88]. An implication of such resilience is that nutrients and building blocks for cellular mass are locked away from the growing fermenting community, slowing down their growth and thus mineralization rates of organic matter.

Our results showed that the oxic and anoxic communities in hadal sediments have different capacities for carbohydrate utilization. The relative abundances of CAZyme families (based upon MAG abundances) showed little variation with sediment depth (see Fig. 4B, Supplementary Fig. 7) until nitrate was entirely depleted. This was true even in the abyssal plains sediments where there was a strong downcore gradient in organic carbon concentration and availability within the oxic zone [18, 29]. This could imply that the relative CAZyme family abundance is not informative of the actual CAZyme activity in seafloor sediments and/or that there is little selective pressure on genomic CAZyme contents.

CAZyme composition and carbohydrate utilization capacity only changed strongly from the top of the ferruginous zone. A possible explanation for the apparent increased importance of CAZyme versatility could be that iron reduction in the ferruginous zone releases carbohydrates bound to reactive iron phases [89], which are abundant in hadal sediments and have been previously shown to bind about one-fifth of organic matter in marine sediments [90]. In the ferruginous zone, the relative abundance of putative complex carbohydrate degraders increased steadily with depth. This suggests that, despite the energetic costs of having a large repertoire of excreted CAZymes, it can still be advantageous in environments where energy is severely limited and where microbes are thought to streamline their genomes to maximize efficiency [9, 10].

Our data highlight the importance of treading carefully when linking changes in microbial community composition to concurrent biogeochemical conditions. Although the sediments of the Atacama trench are inhabited by communities distinct from those of adjacent abyssal sites, our data suggest that it would be a mistake to attribute that distinction to a selective effect of hydrostatic pressure alone—hydrostatic pressure does not appear to be an evolutionary bottleneck overcome by only a few lineages. Furthermore, our data highlight how changes in selective forces do not always co-occur with the shifts in community composition that they ultimately produce, nor do they take the same shapes. In the Atacama trench, redox stratification between the oxic and nitrogenous zones did not impose selective pressure on MAGs based on their respiratory capabilities, as might be expected, but respiratory capability was strongly selected upon in the ferruginous zone. Similarly, carbohydrate degradation potential was not selected upon in the nitrogenous or oxic zones but MAGs with the potential to degrade complex carbohydrates (i.e. with many excreted CAZymes) increased in relative abundance in the ferruginous zone, where we also observed a potential deep-rooting niche differentiation between phyla by CAZyme content. This partial decoupling between biogeochemistry, function, and community structure may be explained by the extremely long generation times of the benthic communities. It takes centuries for the aerobic and nitrogen respiring community to decay and the fermenters to grow, smearing the abrupt transitions between biogeochemical zones into smooth gradients in community composition.

## Supplementary Material

Hadal_metagenomes_supplements_clean_ycad005

## Data Availability

The sequencing data of this study is accessible under the bioproject ID PRJEB57745 on the NCBI short-read archive (SRA).

## References

[1] Hedges JI, Keil RG. Sedimentary organic matter preservation: an assessment and speculative synthesis. Mar Chem 1995;49:81–115. 10.1016/0304-4203(95)00008-F.

[2] Canfield DE . Factors influencing organic carbon preservation in marine sediments. Chem Geol 1994;114:315–29. 11539298 10.1016/0009-2541(94)90061-2

[3] Thullner M, Dale AW, Regnier P. Global-scale quantification of mineralization pathways in marine sediments: a reaction-transport modeling approach. Geochem Geophys Geosystems 2009;10:1–24.

[4] Kallmeyer J, Pockalny R, Adhikari RR et al. Global distribution of microbial abundance and biomass in subseafloor sediment. Proc Natl Acad Sci U S A 2012;109:16213–6. 22927371 10.1073/pnas.1203849109 PMC3479597

[5] Arndt S, Jørgensen BB, LaRowe DE et al. Quantifying the degradation of organic matter in marine sediments: a review and synthesis. Earth Sci Rev 2013;123:53–86.

[6] Froelich PNN, Klinkhammer GPP, Bender MLL et al. Early oxidation of organic matter in pelagic sediments of the eastern equatorial Atlantic: suboxic diagenesis. Geochim Cosmochim Acta 1979;43:1075–90. 10.1016/0016-7037(79)90095-4.

[7] Canfield DE, Thamdrup B. Towards a consistent classification scheme for geochemical environments, or, why we wish the term ‘suboxic’ would go away. Geobiology 2009;7:385–92. 19702823 10.1111/j.1472-4669.2009.00214.x

[8] Petro C, Starnawski P, Schramm A et al. Microbial community assembly in marine sediments. Aquat Microb Ecol 2017;79:177–95.

[9] Starnawski P, Bataillon T, Ettema TJG et al. Microbial community assembly and evolution in subseafloor sediment. Proc Natl Acad Sci U S A 2017;114:2940–5. 10.1073/pnas.1614190114. 28242677 PMC5358386

[10] Lever MA, Rogers KL, Lloyd KG et al. Life under extreme energy limitation: a synthesis of laboratory- and field-based investigations. FEMS Microbiol Rev 2015;39:688–728. 25994609 10.1093/femsre/fuv020

[11] Stewart HA, Jamieson AJ. Habitat heterogeneity of hadal trenches: considerations and implications for future studies. Prog Oceanogr 2018;161:47–65.

[12] Jamieson A . *The Hadal Zone*: *Life in the Deepest Oceans*. Cambridge, England: Cambridge University Press, 2015.

[13] Jamieson AJ, Fujii T, Mayor DJ et al. Hadal trenches: the ecology of the deepest places on Earth. Trends Ecol Evol 2010;25:190–7. 19846236 10.1016/j.tree.2009.09.009

[14] Oguri K, Masqué P, Zabel M et al. Sediment accumulation and carbon burial in four hadal trench systems. J Geophys Res Biogeosci 2022;127:e2022JG006814. 10.1029/2022JG006814.

[15] Kioka A, Schwestermann T, Moernaut J et al. Megathrust earthquake drives drastic organic carbon supply to the hadal trench. Sci Rep 2019;9:1553. 30733607 10.1038/s41598-019-38834-x PMC6367409

[16] Turnewitsch R, Falahat S, Stehlikova J et al. Recent sediment dynamics in hadal trenches: evidence for the influence of higher-frequency (tidal, near-inertial) fluid dynamics. Deep Sea Res I 2014;90:125–38. 10.1016/j.dsr.2014.05.005.

[17] Glud RN, Wenzhofer F, Middelboe M et al. High rates of microbial carbon turnover in sediments in the deepest oceanic trench on Earth. Nat Geosci 2013;6:284–8. 10.1038/ngeo1773.

[18] Glud RN, Berg P, Thamdrup B et al. Hadal trenches are dynamic hotspots for early diagenesis in the deep sea. Commun Earth Environ 2021;2:1–8. 10.1038/s43247-020-00087-2.

[19] Thamdrup B, Schauberger C, Larsen M et al. Anammox bacteria drive fixed nitrogen loss in hadal trench sediments. Proc Natl Acad Sci U S A 2021;118:e2104529118. 34764222 10.1073/pnas.2104529118 PMC8609620

[20] Thamdrup B, Schauberger C, Wenzhöfer F et al. Anaerobic Carbon Mineralization in Hadal Sediments. Goldschmidt2021• Virtual• 4-9 July 2021.

[21] Petro C, Zäncker B, Starnawski P et al. Marine deep biosphere microbial communities assemble in near-surface sediments in Aarhus Bay. Front Microbiol 2019;10:758. 31031732 10.3389/fmicb.2019.00758 PMC6474314

[22] Jamieson AJ, Weston JNJ. Amphipoda from depths exceeding 6,000 meters revisited 60 years on. J Crustac Biol 2023;43:ruad020.

[23] Schauberger C, Seki D, Cutts EM et al. Uniform selective pressures within redox zones drive gradual changes in microbial community composition in hadal sediments. Environ Microbiol 2023;25:1594–604. 36999247 10.1111/1462-2920.16377

[24] Peoples LM, Grammatopoulou E, Pombrol M et al. Microbial community diversity within sediments from two geographically separated hadal trenches. Front Microbiol 2019;10:347. 10.3389/fmicb.2019.00347. PMC6428765 30930856

[25] Hiraoka S, Hirai M, Matsui Y et al. Microbial community and geochemical analyses of trans-trench sediments for understanding the roles of hadal environments. ISME J 2020;14:740–56. 10.1038/s41396-019-0564-z. 31827245 PMC7031335

[26] Zhang X, Xu W, Liu Y et al. Metagenomics reveals microbial diversity and metabolic potentials of seawater and surface sediment from a hadal biosphere at the Yap Trench. Front Microbiol 2018;9:2402. 10.3389/fmicb.2018.02402. PMC6194347 30369913

[27] Zhou YL, Mara P, Cui GJ et al. Microbiomes in the challenger deep slope and bottom-axis sediments. Nat Commun 2022;13:1–13. 35314706 10.1038/s41467-022-29144-4 PMC8938466

[28] Schauberger C, Glud RN, Hausmann B et al. Microbial community structure in hadal sediments: high similarity along trench axes and strong changes along redox gradients. ISME J 2021;15:3455–67. 34103697 10.1038/s41396-021-01021-w PMC8629969

[29] Schauberger C, Middelboe M, Larsen M et al. Spatial variability of prokaryotic and viral abundances in the Kermadec and Atacama Trench regions. Limnol Oceanogr 2021;66:2095–109. 34239169 10.1002/lno.11711 PMC8248377

[30] Wasmund K, Pelikan C, Schintlmeister A et al. Genomic insights into diverse bacterial taxa that degrade extracellular DNA in marine sediments. Nat Microbiol 2021;6:885–98. 34127845 10.1038/s41564-021-00917-9 PMC8289736

[31] Pelikan C, Wasmund K, Glombitza C et al. Anaerobic bacterial degradation of protein and lipid macromolecules in subarctic marine sediment. ISME J 2021;15:833–47. 33208892 10.1038/s41396-020-00817-6 PMC8027456

[32] Paul R, Rogers TJ, Fullerton KM et al. Complex organic matter degradation by secondary consumers in chemolithoautotrophy-based subsurface geothermal ecosystems. PLoS One 2023;18:e0281277. 37594978 10.1371/journal.pone.0281277 PMC10437873

[33] Lombard V, Golaconda Ramulu H, Drula E et al. The carbohydrate-active enzymes database (CAZy) in 2013. Nucleic Acids Res 2014;42:D490–5. 24270786 10.1093/nar/gkt1178 PMC3965031

[34] Dutschei T, Beidler I, Bartosik D et al. Marine *Bacteroidetes* enzymatically digest xylans from terrestrial plants. Environ Microbiol 2023;25:1713–27. 37121608 10.1111/1462-2920.16390

[35] Thamdrup B, Schauberger C, Larsen M et al. Benthic nitrogen cycling in hadal trenches: high rates and large contributions from anammox. Ocean Sci Meet 2020.

[36] Eren AM, Vineis JH, Morrison HG et al. A filtering method to generate high quality short reads using Illumina paired-end technology. PLoS One 2013;8:e66643. 23799126 10.1371/journal.pone.0066643 PMC3684618

[37] Minoche AE, Dohm JC, Himmelbauer H. Evaluation of genomic high-throughput sequencing data generated on Illumina HiSeq and Genome Analyzer systems. Genome Biol 2011;12:R112. 22067484 10.1186/gb-2011-12-11-r112 PMC3334598

[38] Shaiber A, Willis AD, Delmont TO et al. Functional and genetic markers of niche partitioning among enigmatic members of the human oral microbiome. Genome Biol 2020;21:1–35. 10.1186/s13059-020-02195-w PMC7739484 33323122

[39] Langmead B, Salzberg SL. Fast gapped-read alignment with bowtie 2. Nat Methods 2012;9:357–9. 22388286 10.1038/nmeth.1923 PMC3322381

[40] Alneberg J, Bjarnason BS, de Bruijn I et al. Binning metagenomic contigs by coverage and composition. Nat Methods 2014;11:1144–6. 25218180 10.1038/nmeth.3103

[41] Kang DD, Li F, Kirton E et al. MetaBAT 2: an adaptive binning algorithm for robust and efficient genome reconstruction from metagenome assemblies. PeerJ 2019;7:e7359. 31388474 10.7717/peerj.7359 PMC6662567

[42] Wu Y-W, Simmons BA, Singer SW. MaxBin 2.0: an automated binning algorithm to recover genomes from multiple metagenomic datasets. Bioinformatics 2016;32:605–7. 26515820 10.1093/bioinformatics/btv638

[43] Sieber CMK, Probst AJ, Sharrar A et al. Recovery of genomes from metagenomes via a dereplication, aggregation and scoring strategy. Nat Microbiol 2018;3:836–43. 29807988 10.1038/s41564-018-0171-1 PMC6786971

[44] Parks DH, Imelfort M, Skennerton CT et al. CheckM: assessing the quality of microbial genomes recovered from isolates, single cells, and metagenomes. Genome Res 2015;25:1043–55. 25977477 10.1101/gr.186072.114 PMC4484387

[45] Olm MR, Brown CT, Brooks B et al. dRep: a tool for fast and accurate genomic comparisons that enables improved genome recovery from metagenomes through de-replication. ISME J 2017;11:2864–8. 28742071 10.1038/ismej.2017.126 PMC5702732

[46] Eren AM, Kiefl E, Shaiber A et al. Community-led, integrated, reproducible multi-omics with anvi’o. Nat Microbiol 2021;6:3–6. 10.1038/s41564-020-00834-3. 33349678 PMC8116326

[47] Lee MD . GToTree: a user-friendly workflow for phylogenomics. Bioinformatics 2019;35:4162–4. 30865266 10.1093/bioinformatics/btz188 PMC6792077

[48] Chaumeil P-A, Mussig AJ, Hugenholtz P et al. GTDB-Tk: a toolkit to classify genomes with the genome taxonomy database. Bioinformatics 2020;36:1925–7. 10.1093/bioinformatics/btz848 PMC7703759 31730192

[49] Parks DH, Chuvochina M, Rinke C et al. GTDB: an ongoing census of bacterial and archaeal diversity through a phylogenetically consistent, rank normalized and complete genome-based taxonomy. Nucleic Acids Res 2022;50:D785–94. 34520557 10.1093/nar/gkab776 PMC8728215

[50] Minh BQ, Schmidt HA, Chernomor O et al. IQ-TREE 2: new models and efficient methods for phylogenetic inference in the genomic era. Mol Biol Evol 2020;37:1530–4. 32011700 10.1093/molbev/msaa015 PMC7182206

[51] Kalyaanamoorthy S, Minh BQ, Wong TKF et al. ModelFinder: fast model selection for accurate phylogenetic estimates. Nat Methods 2017;14:587–9. 28481363 10.1038/nmeth.4285 PMC5453245

[52] Hoang DT, Chernomor O, von Haeseler A et al. UFBoot2: improving the ultrafast bootstrap approximation. Mol Biol Evol 2018;35:518–22. 29077904 10.1093/molbev/msx281 PMC5850222

[53] Guindon S, Dufayard J-F, Lefort V et al. New algorithms and methods to estimate maximum-likelihood phylogenies: assessing the performance of PhyML 3.0. Syst Biol 2010;59:307–21. 20525638 10.1093/sysbio/syq010

[54] Letunic I, Bork P. Interactive Tree Of Life (iTOL) v5: an online tool for phylogenetic tree display and annotation. Nucleic Acids Res 2021;49:W293–6. 33885785 10.1093/nar/gkab301 PMC8265157

[55] R Core Team . R: A Language and Environment for Statistical Computing. Vienna, Austria: R Foundation for Statistical Computing, 2022.

[56] Kembel SW, Cowan PD, Helmus MR et al. Picante: R tools for integrating phylogenies and ecology. Bioinformatics 2010;26:1463–4. 20395285 10.1093/bioinformatics/btq166

[57] Hyatt D, Chen G-L, LoCascio PF et al. Prodigal: prokaryotic gene recognition and translation initiation site identification. BMC Bioinformatics 2010;11:119. 20211023 10.1186/1471-2105-11-119 PMC2848648

[58] Aramaki T, Blanc-Mathieu R, Endo H et al. KofamKOALA: KEGG ortholog assignment based on profile HMM and adaptive score threshold. Bioinformatics 2020;36:2251–2. 31742321 10.1093/bioinformatics/btz859 PMC7141845

[59] Cantalapiedra CP, Hernández-Plaza A, Letunic I et al. eggNOG-mapper v2: functional annotation, orthology assignments, and domain prediction at the metagenomic scale. Mol Biol Evol 2021;38:5825–9. 34597405 10.1093/molbev/msab293 PMC8662613

[60] Garber AI, Nealson KH, Okamoto A et al. FeGenie: a comprehensive tool for the identification of iron genes and iron gene neighborhoods in genome and metagenome assemblies. Front Microbiol 2020;11:37. 32082281 10.3389/fmicb.2020.00037 PMC7005843

[61] Teufel F, Almagro Armenteros JJ, Johansen AR et al. SignalP 6.0 predicts all five types of signal peptides using protein language models. Nat Biotechnol 2022;40:1023–5. 10.1038/s41587-021-01156-3. 34980915 PMC9287161

[62] Wickham H, Averick M, Bryan J et al. Welcome to the Tidyverse. J Open Source Softw 2019;4:1686.

[63] Gao C-H, Yu G, Cai P. ggVennDiagram: an intuitive, easy-to-use, and highly customizable R package to generate Venn diagram. Front Genet 2021;12:1598. 10.3389/fgene.2021.706907. PMC8452859 34557218

[64] Alboukadel K . *ggpubr*: *‘ggplot2’ Based Publication Ready Plots*. 2023.

[65] Garnier S, Ross N, Rudis Bo B et al. *Sjmgarnier/Viridis*: *CRAN Release v0.6.2*. Zenodo, 2021. 10.5281/zenodo.5579397.

[66] Dowle M, Srinivasan A. *data.table*: *Extension of `data.frame*`. 2023.

[67] Andersen K, Kirkegaard R, Karst S et al. ampvis2: an R package to analyse and visualise 16S rRNA amplicon data. BioRxiv 2018;299537.

[68] Oksanen J, Blanchet FG, Friendly M et al. Package ‘vegan’ title community ecology package. Commun Ecol Package 2019;2:1–297.

[69] Kerou M, Offre P, Valledor L et al. Proteomics and comparative genomics of *Nitrososphaera viennensis* reveal the core genome and adaptations of archaeal ammonia oxidizers. Proc Natl Acad Sci U S A 2016;113:E7937-E7946. 10.1073/pnas.1601212113. PMC5150414 27864514

[70] Bueno E, Mesa S, Bedmar EJ et al. Bacterial adaptation of respiration from oxic to microoxic and anoxic conditions: redox control. Antioxid Redox Signal 2012;16:819–52. 22098259 10.1089/ars.2011.4051 PMC3283443

[71] Wasmund K, Mußmann M, Loy A. The life sulfuric: microbial ecology of sulfur cycling in marine sediments. Environ Microbiol Rep 2017;9:323–44. 28419734 10.1111/1758-2229.12538 PMC5573963

[72] Hashimoto H . Recent structural studies of carbohydrate-binding modules. Cell Mol Life Sci 2006;63:2954–67. 17131061 10.1007/s00018-006-6195-3 PMC11136102

[73] Garron M-L, Henrissat B. The continuing expansion of CAZymes and their families. Curr Opin Chem Biol 2019;53:82–7. 31550558 10.1016/j.cbpa.2019.08.004

[74] Dik DA, Marous DR, Fisher JF et al. Lytic transglycosylases: concinnity in concision of the bacterial cell wall. Crit Rev Biochem Mol Biol 2017;52:503–42. 28644060 10.1080/10409238.2017.1337705 PMC6102726

[75] Costa OYA, de Hollander M, Pijl A et al. Cultivation-independent and cultivation-dependent metagenomes reveal genetic and enzymatic potential of microbial community involved in the degradation of a complex microbial polymer. Microbiome 2020;8:76. 32482164 10.1186/s40168-020-00836-7 PMC7265232

[76] Henrissat B, Davies G. Structural and sequence-based classification of glycoside hydrolases. Curr Opin Struct Biol 1997;7:637–44. 9345621 10.1016/s0959-440x(97)80072-3

[77] Cutts EM, Baldes MJ, Skoog EJ et al. Using molecular tools to understand microbial carbonates. Geosciences 2022;12:185.

[78] Zhao R, Summers ZM, Christman GD et al. Metagenomic views of microbial dynamics influenced by hydrocarbon seepage in sediments of the Gulf of Mexico. Sci Rep 2020;10:5772. 32238866 10.1038/s41598-020-62840-z PMC7113308

[79] Bienhold C, Zinger L, Boetius A et al. Diversity and biogeography of bathyal and abyssal seafloor bacteria. PLoS One 2016;11:e0148016. 26814838 10.1371/journal.pone.0148016 PMC4731391

[80] Sunagawa S, Coelho LP, Chaffron S et al. Structure and function of the global ocean microbiome. Science 2015;348:1261359. 10.1126/science.1261359. 25999513

[81] Zabel M, Glud RN, Sanei H et al. High carbon mineralization rates in subseafloor hadal sediments—result of frequent mass wasting. Geochem Geophys Geosystems 2022;23:e2022GC010502.

[82] Simon J, Klotz MG. Diversity and evolution of bioenergetic systems involved in microbial nitrogen compound transformations. Biochim Biophys Acta Bioenerg 2013;1827:114–35. 10.1016/j.bbabio.2012.07.005 22842521

[83] Chain PSG, Grafham DV, Fulton RS et al. Genome project standards in a new era of sequencing. Science 2009;326:236–7. 19815760 10.1126/science.1180614 PMC3854948

[84] Temme HR, Carlson A, Novak PJ. Presence, diversity, and enrichment of respiratory reductive dehalogenase and non-respiratory hydrolytic and oxidative dehalogenase genes in terrestrial environments. Front Microbiol 2019;10:1258. 31231342 10.3389/fmicb.2019.01258 PMC6567934

[85] Kessler AJ, Chen YJ, Waite DW et al. Bacterial fermentation and respiration processes are uncoupled in anoxic permeable sediments. Nat Microbiol 2019;4:1014–23. 30858573 10.1038/s41564-019-0391-z

[86] Fiore CL, Jarett JK, Olson ND et al. Nitrogen fixation and nitrogen transformations in marine symbioses. Trends Microbiol 2010;18:455–63. 20674366 10.1016/j.tim.2010.07.001

[87] Durbin AM, Teske A. Microbial diversity and stratification of South Pacific abyssal marine sediments. Environ Microbiol 2011;13:3219–34. 21895908 10.1111/j.1462-2920.2011.02544.x

[88] Jochum LM, Chen X, Lever MA et al. Depth distribution and assembly of sulfatereducing microbial communities in marine sediments of Aarhus Bay. Appl Environ Microbiol 2017;83:e01547–17. 28939599 10.1128/AEM.01547-17 PMC5691419

[89] Aftabtalab A, Rinklebe J, Shaheen SM et al. Review on the interactions of arsenic, iron (oxy)(hydr)oxides, and dissolved organic matter in soils, sediments, and groundwater in a ternary system. Chemosphere 2022;286:131790. 34388870 10.1016/j.chemosphere.2021.131790

[90] Lalonde K, Mucci A, Ouellet A et al. Preservation of organic matter in sediments promoted by iron. Nature 2012;483:198–200. 22398559 10.1038/nature10855

