# Supplementary material for: Metagenome-assembled genomes of deep-sea sediments: changes in microbial functional potential lag behind redox transitions
Source: ISME Commun. 2024 Jan 10;4(1):ycad005. doi: 10.1093/ismeco/ycad005 (PMC10809760; doi:10.1093/ismeco/ycad005)
Supplement: Hadal_metagenomes_supplements_clean_ycad005 [file hadal_metagenomes_supplements_clean_ycad005.docx]

**Supplementary information for**

**Metagenome-assembled genomes of deep-sea sediments: changes in microbial functional potential lag behind redox transitions**

Clemens Schauberger^1*^, Bo Thamdrup^1^, Clarisse Lemonnier^2^, Blandine Trouche^2^, Julie Poulain^3^, Patrick Wincker^3^, Sophie Arnaud-Haond^4^, Ronnie N. Glud^1,5,^ Lois Maignien^2^

**Affiliation:**

^1^ Department of Biology, University of Southern Denmark, 5230 Odense M, Denmark

^2^ Univ Brest, CNRS, IFREMER, Microbiology of Extreme Environments Laboratory, F-29280 Plouzané, France

^3^ Génomique Métabolique, Genoscope, Institut François Jacob, CEA, CNRS, Univ Evry, Université Paris-Saclay, Evry, France

^4^ MARBEC, Institut Français de Recherche pour L'Exploitation de la Mer, Univ Montpellier, CNRS, IRD, Sète, France

^5^ Department of Ocean and Environmental Sciences, Tokyo University of Marine Science and Technology, Tokyo, Japan

**Supplementary Material and Methods:**

Hydrogen sulfide was measured in porewater extracts from parallel cores (porewater extracts from Thamdrup *et al*., 2021). In brief, 9.4 cm inner diameter Plexiglas sediment cores were immediately transferred to a 3°C cold room after retrieval. Core sectioning typically started within one hour of sample collection within a nitrogen-filled glove bag. Porewater was extracted through centrifugation, and samples were fixed with a 1% weight-to-volume ratio of zinc acetate (1:100 vol:vol) and subsequently analyzed spectrophotometrically following the method described by Cline (1969).

Sulfate reduction rates were determined in intact cores according to Jørgensen (1978). This involved injecting approximately 40 kBq of carrier-free ^35^S-SO_4_^2-^ at 1 cm intervals, followed by a 24-hour incubation at 3°C. Cores were then sliced at 2 cm intervals, preserved by mixing with a 20% weight-to-volume ratio of zinc acetate (1:1 vol:vol), and frozen. The quantification of ^35^S in the total reduced inorganic sulfur (TRIS) pool was carried out through distillation with hot acidic Cr^2+^ after washing the sediment pellet to remove ^35^S-SO_4_^2-^, as described by Canfield *et al*. (1986) and Fossing and Jørgensen (1989).

**References:**

Canfield, D. E., Raiswell, R., Westrich, J. T., Reaves, C. M., & Berner, R. A. (1986). The use of chromium reduction in the analysis of reduced inorganic sulfur in sediments and shales. *Chemical geology*, *54*(1-2), 149-155.

Cline, J. D. (1969). Spectrophotometric determination of hydrogen sulfide in natural waters 1. *Limnology and Oceanography*, *14*(3), 454-458.

Fossing, H., & Jørgensen, B. B. (1989). Measurement of bacterial sulfate reduction in sediments: evaluation of a single-step chromium reduction method. *Biogeochemistry*, *8*, 205-222.

Glud, R. N., Berg, P., Thamdrup, B., Larsen, M., Stewart, H. A., Jamieson, A. J., ... & Wenzhöfer, F. (2021). Hadal trenches are dynamic hotspots for early diagenesis in the deep sea. *Communications Earth & Environment*, *2*(1), 21.

Jorgensen, B. B. (1978). A comparison of methods for the quantification of bacterial sulfate reduction in coastal marine sediments. *Geomicrobiology Journal*, *1*(1), 11-27.

**Supplementary Tables:**

**Supplement Table 1:** Phylogenetic diversity calculations and overview of the bacterial tree.

**Supplement Table 2:** Phylogenetic diversity calculations and overview of the archaeal tree.

**Supplement Table 3: Overview over hydrogen sulfide and sulfate reduction rate measurements.**

**Supplementary Figures:**

**
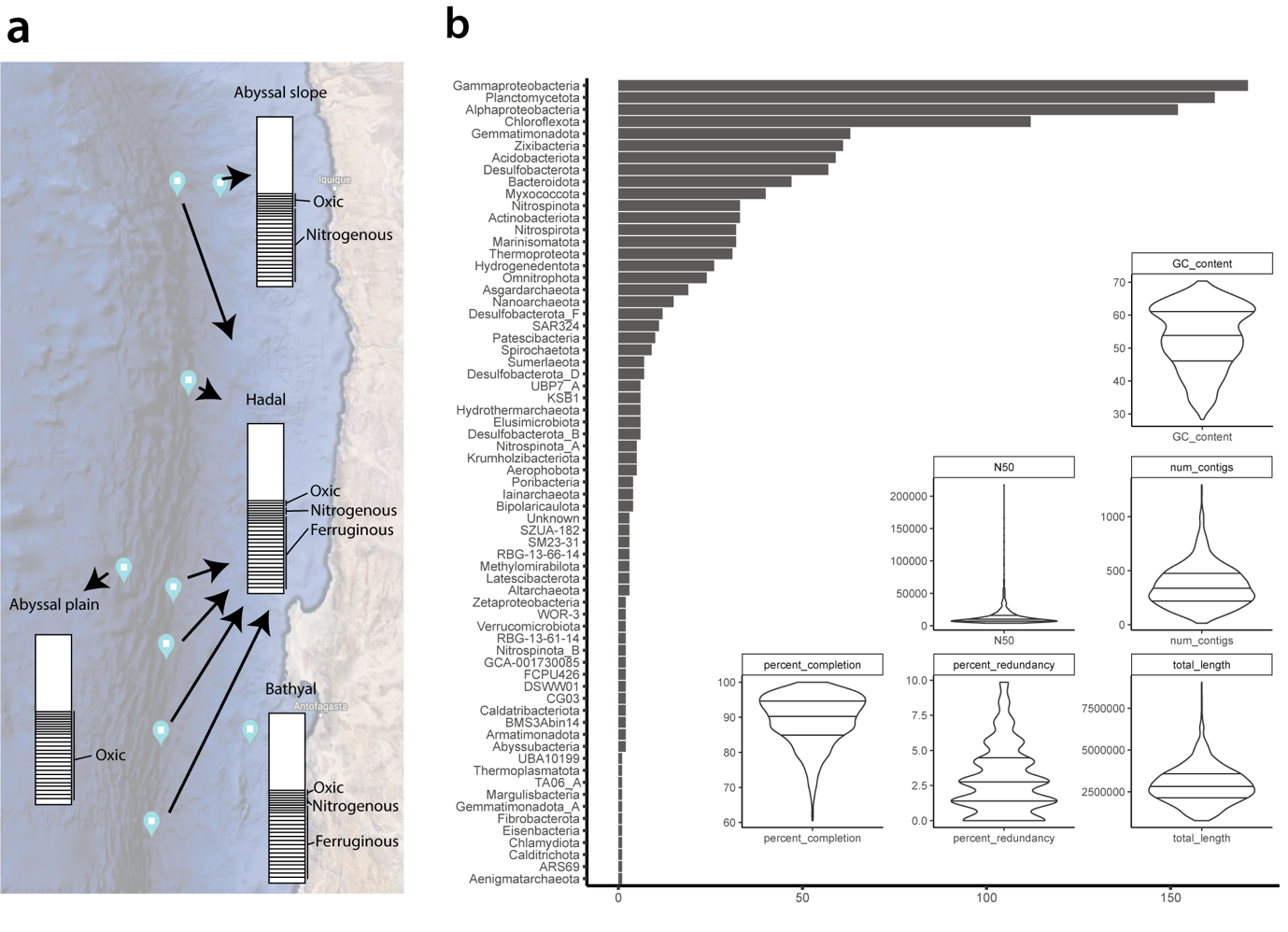
**

**Supplement Figure 1:** **a-b** Schematic map of sampling locations and an overview of basic MAG information. **(a)** The map (Google Maps, 2021) displays the sampling locations along the Chilian coastline. The schematic sediment cores (≤ 45 cm) with line densities represent the sediment sectioning scheme (1 – 2.5 cm), with annotations on the side indicating the approximate biogeochemical zonations within each site. **(b)** Barplot showing the number of MAGs obtained from each phylum (Proteobacteria:Class). The six violin plots within the barplot display the GC contents, N50 values, number of contigs, estimated percent completions and redundancies (contamination), as well as the genome lengths of the MAGs.

**
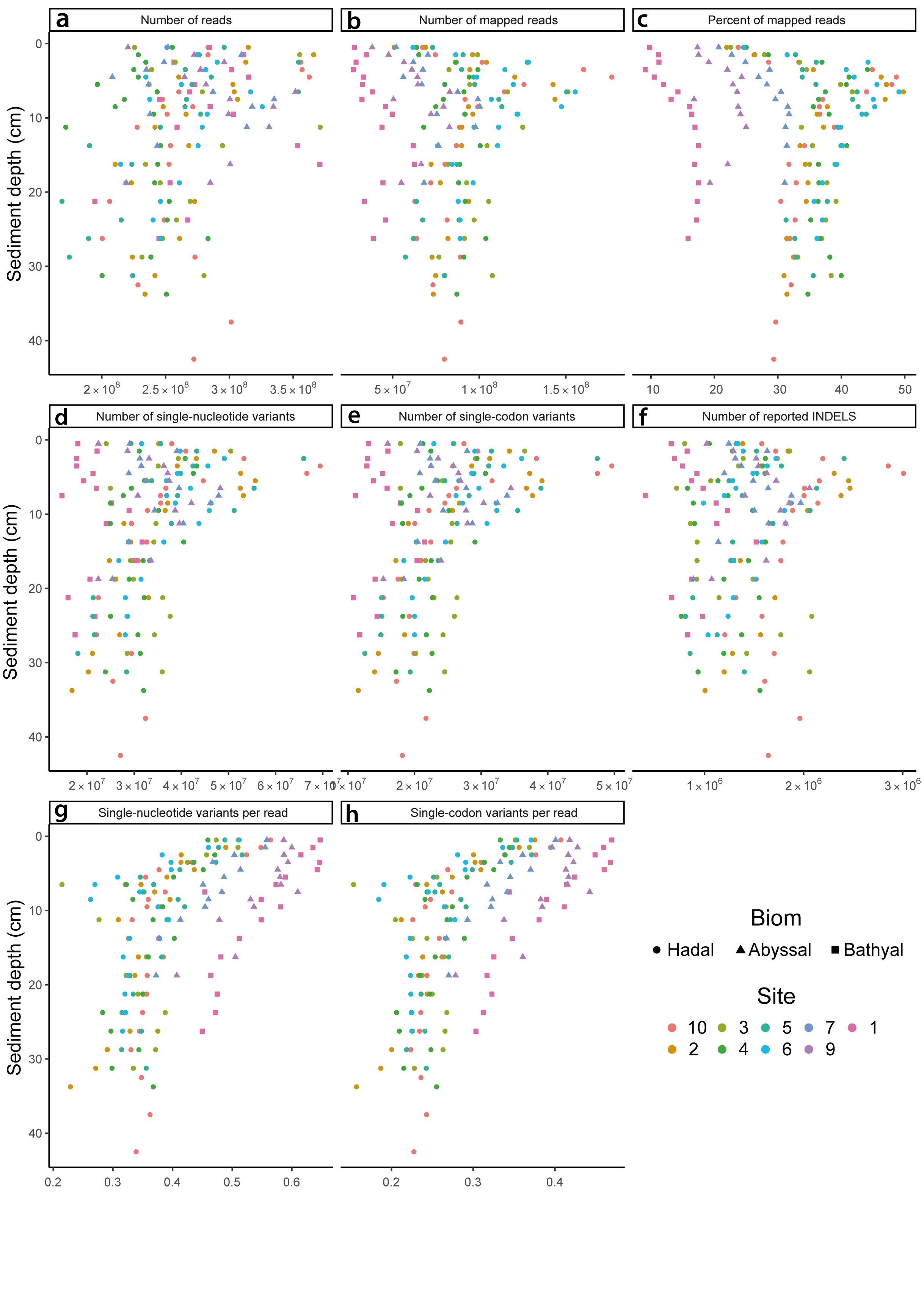
**

**Supplement Figure 2:** **a-h** Dot plots of metagenomic metrics plotted against sediment depth. The nine panels show the **(a)** number of reads, **(b)** number of mapped reads, **(c)** percentage of mapped reads, **(d)** number of single nucleotide variants (SNVs) reported, **(e)** number of single codon variants (SCVs) reported **(f)** number of small insertions and deletions (INDELs) reported, **(g)** number of SNVs per read, and **(h)** number of SCVs per read. Each dot represents the metric a sample and was plotted against sediment depth (y axis), with different colors indicating different sampling sites and shapes indicating the oceanic realm from which the sample was obtained.


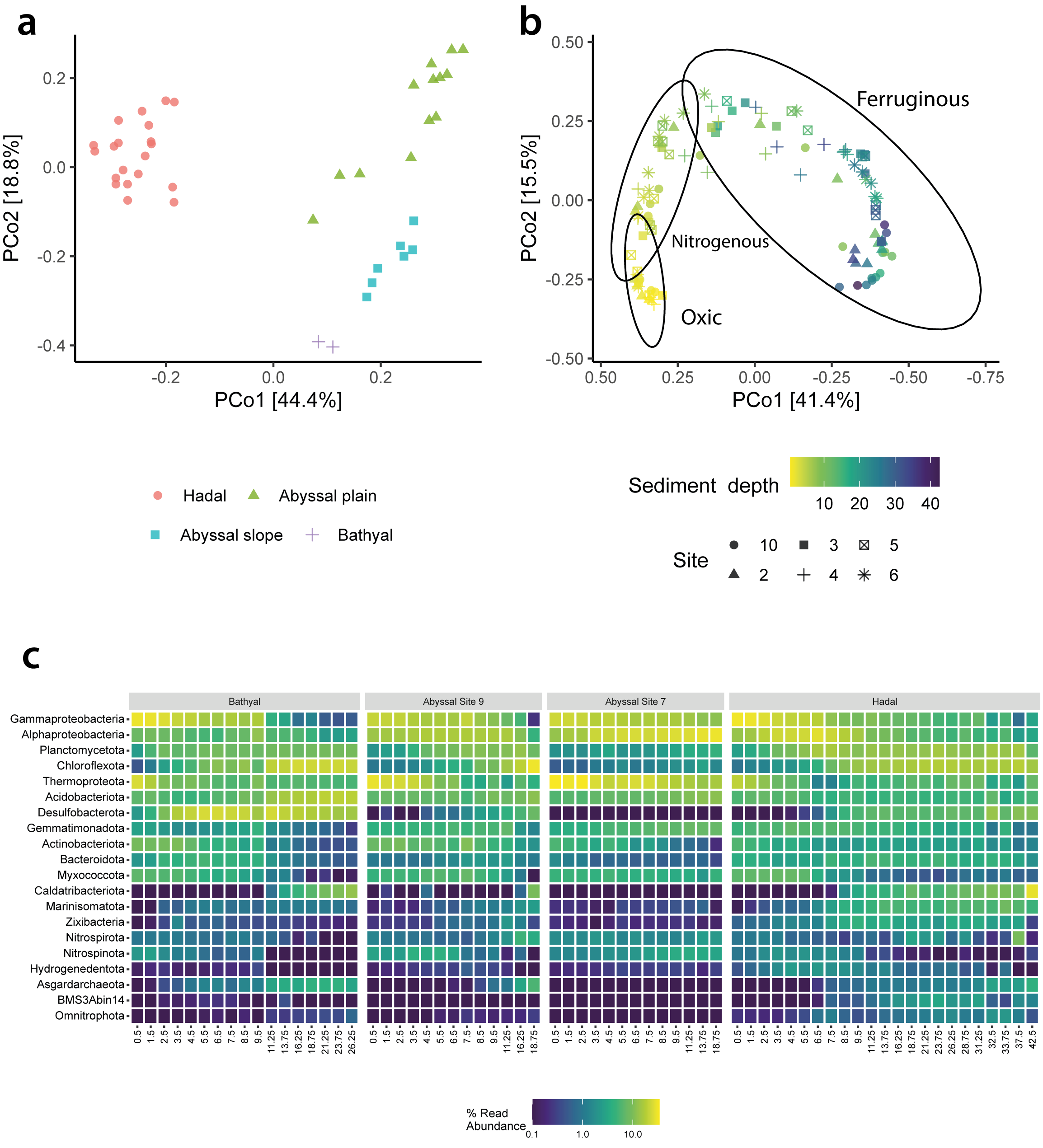


**Supplement Figure 3:** **a,** Principal coordinate analysis (PCOA) of Bray-Curtis dissimilarity, comparing microbial communities from oxic zones of hadal, abyssal plain, abyssal slope, and bathyal sediments. The color and shape of each data point represent the sampling site. **b,** PCOA of Bray-Curtis dissimilarity for hadal samples only, with the color gradient indicating sediment depth and the symbols indicating sampling site. **c,** Heatmap displaying the relative abundances of microbial phyla across sediment depths in bathyal, abyssal slope, abyssal plain, and hadal sediments. The left pivot plot shows data from bathyal depths, while the middle plots show data from abyssal slope and abyssal plain, respectively, while the right pivot plot shows the mean relative abundance of phyla (and proteobacterial classes) across six hadal sites.


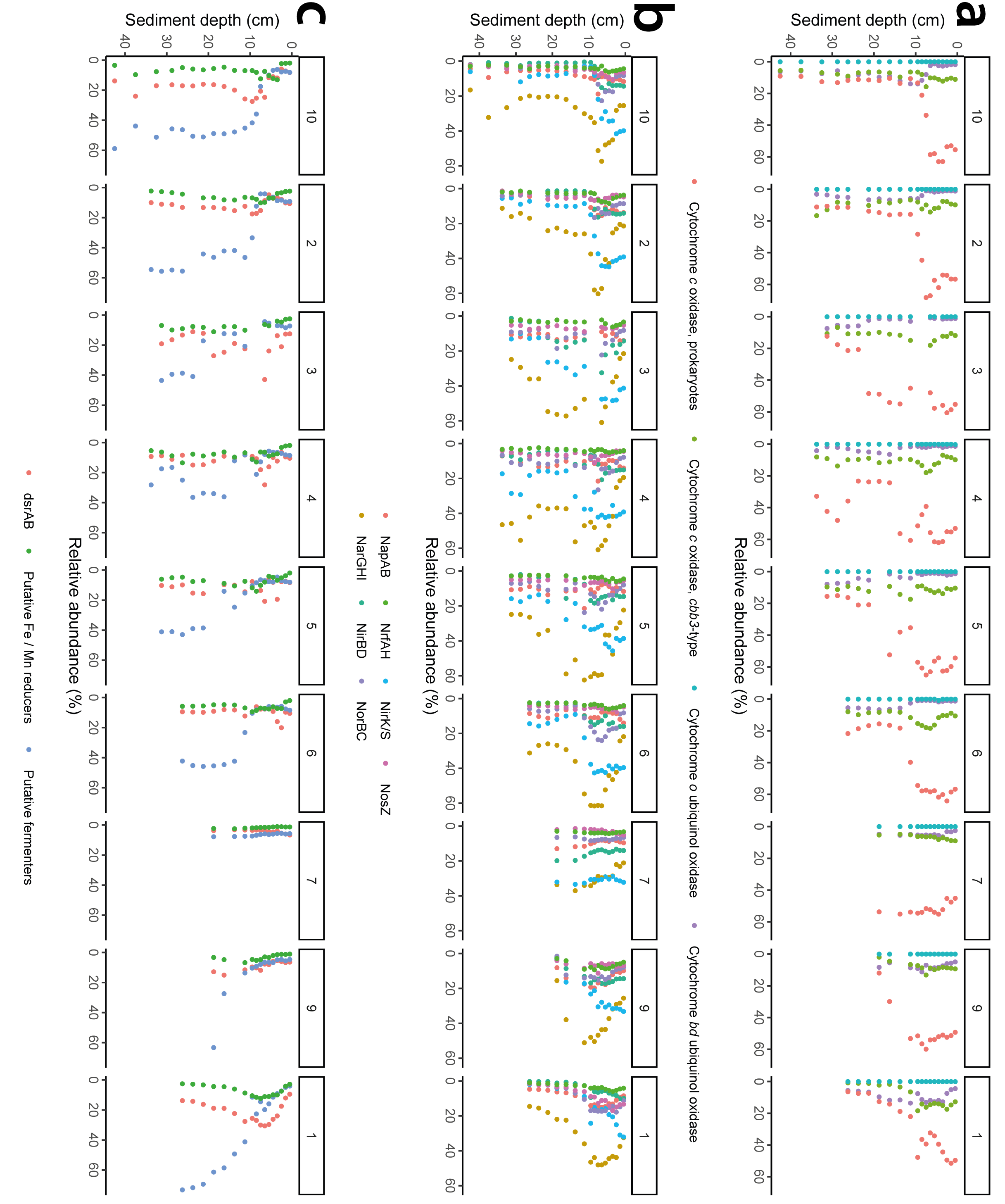


**Supplement Figure 4:** **a-c,** Respiratory trends over sediment depth. **a,** Relative abundances of metagenome-assembled genomes (MAGs) possessing genes for aerobic respiration, including cytochrome *c* oxidase of prokaryotes, cytochrome *c* oxidase *cbb3*-type, cytochrome *o* ubiquinol oxidase, and cytochrome *bd* ubiquinol oxidase, across sediment depth within each sampling site. **b,** Relative abundances of MAGs possessing key enzymes for nitrogen respiration, including napAB, narGHI, nrfAH, nirBD, nirK/S, norBC, and nosZ, across sediment depth within each sampling site. **c,** Relative abundances of MAGs possessing dsrAB, putative Fe/Mn respiration genes, and those without any respiratory capabilities across sediment depth within each sampling site. Each panel includes eight pivot plots, one for each sampling site, depicting the relative abundances of MAGs with specific respiratory genes or capabilities.


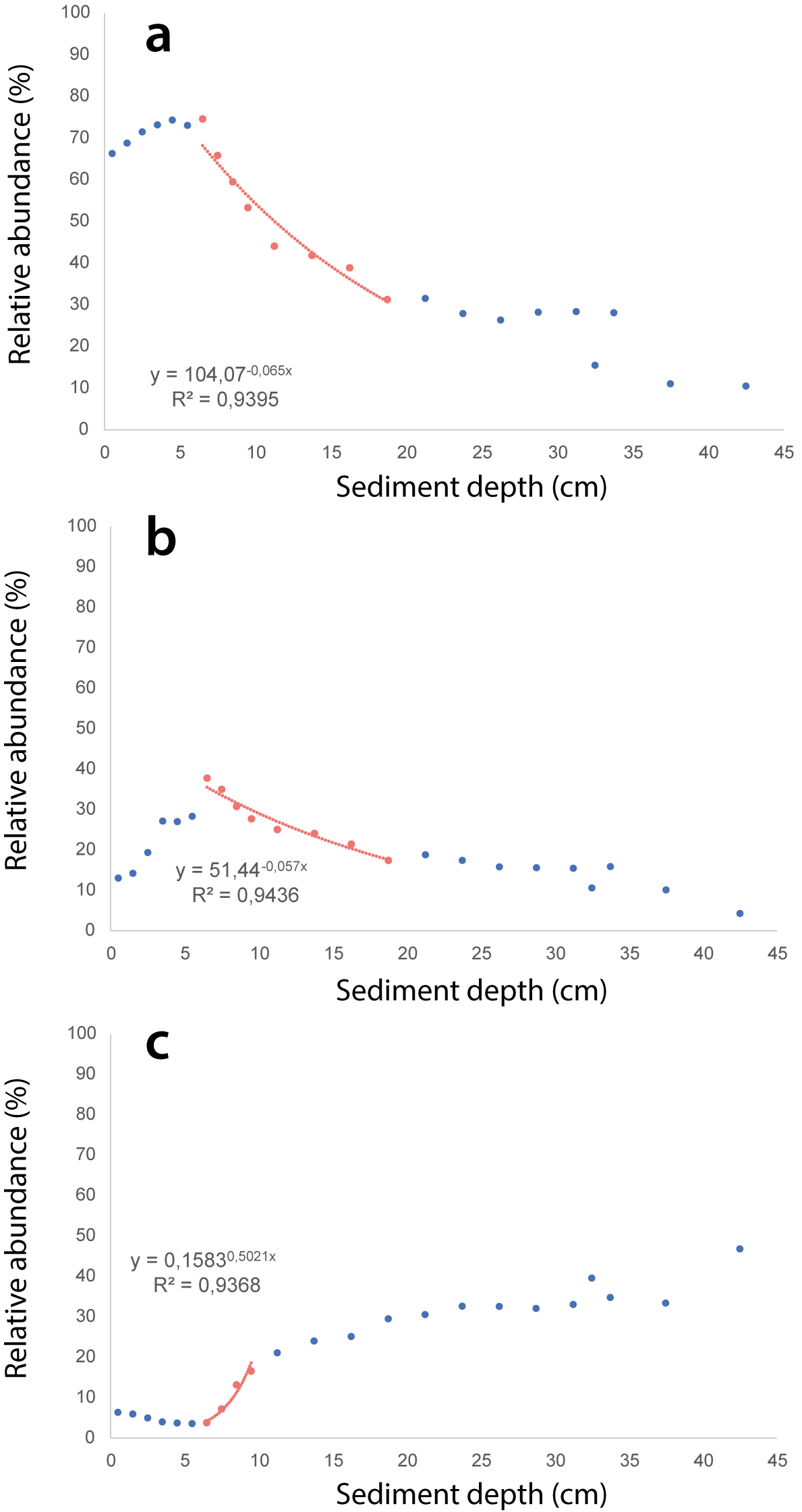


**Supplement Figure 5: a-c,** Analysis of potential half-lifetimes and growth rates for aerobic, nitrogen-respiring, and putative fermenting microorganisms. Dot plots depicting the relative abundance profiles against sediment depth for microbial genomes containing cytochrome *c* oxidases **(a)**, narGHI genes **(b)**, and putative fermenting microorganisms **(c)**. Red dots denote data utilized for estimating growth and half-life times, while blue dots represent the rest of the data. R² values and exponential functions were generated using the least squares method.


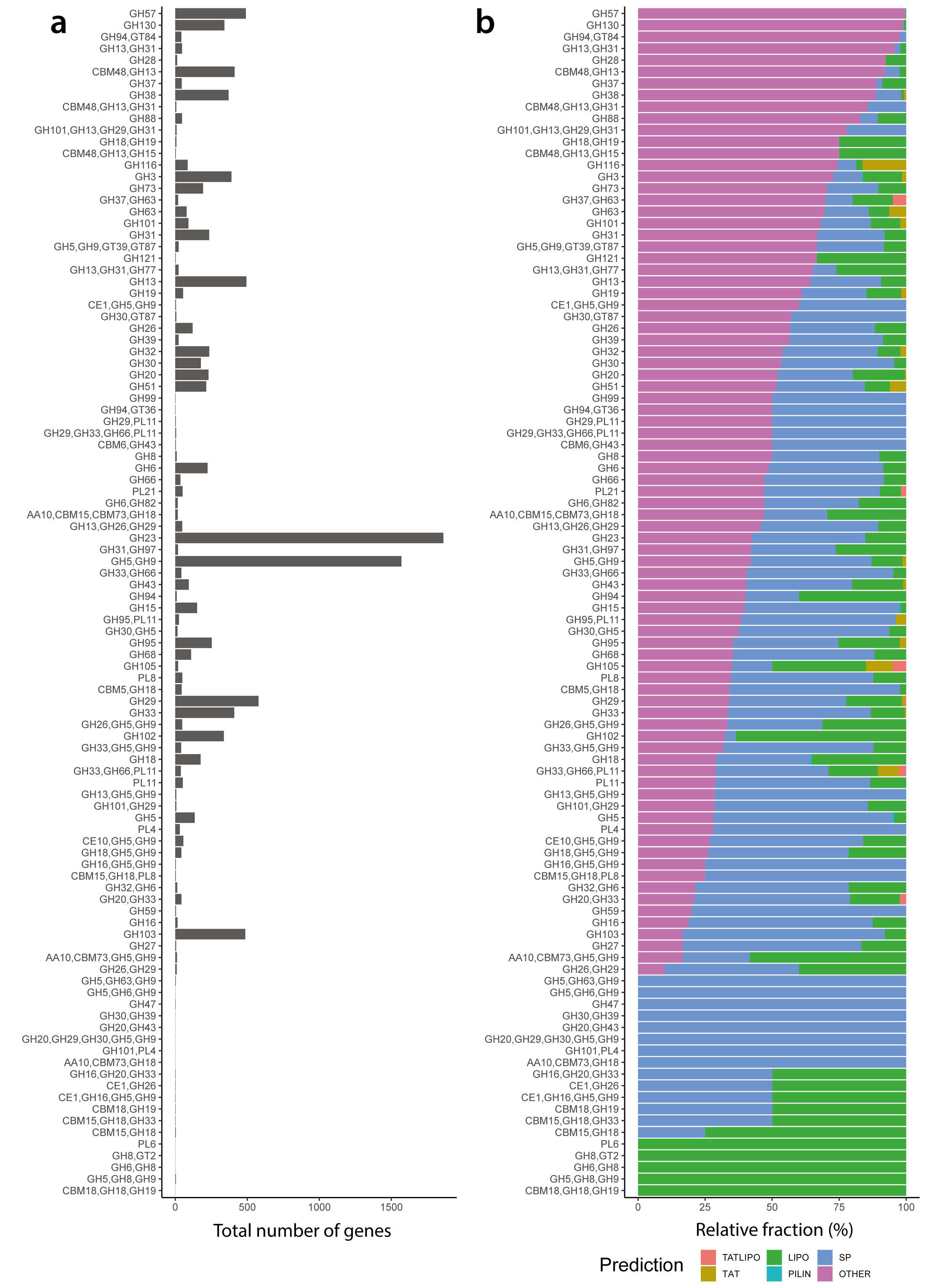


**Supplement Figure 6: a-b,** Carbohydrate-active enzyme (CAZyme) gene family clusters and signal peptide analysis. **a,** barplot displaying the total number of CAZyme genes from each unique CAZyme family clusters. The y-axis shows the different CAZyme clusters, while the x-axis represents the total number of genes for each cluster. **b,** Stacked bar plot depicting the percentage of CAZyme genes with signal peptides for excretion via the TATLIPO, TAT, LIPO, PILIN, SP mechanisms, or without identified signal peptides. The different colors in the plot represent the different excretion mechanisms, with the height of each bar representing the total percentage of genes with or without signal peptides for each unique CAZyme family cluster.


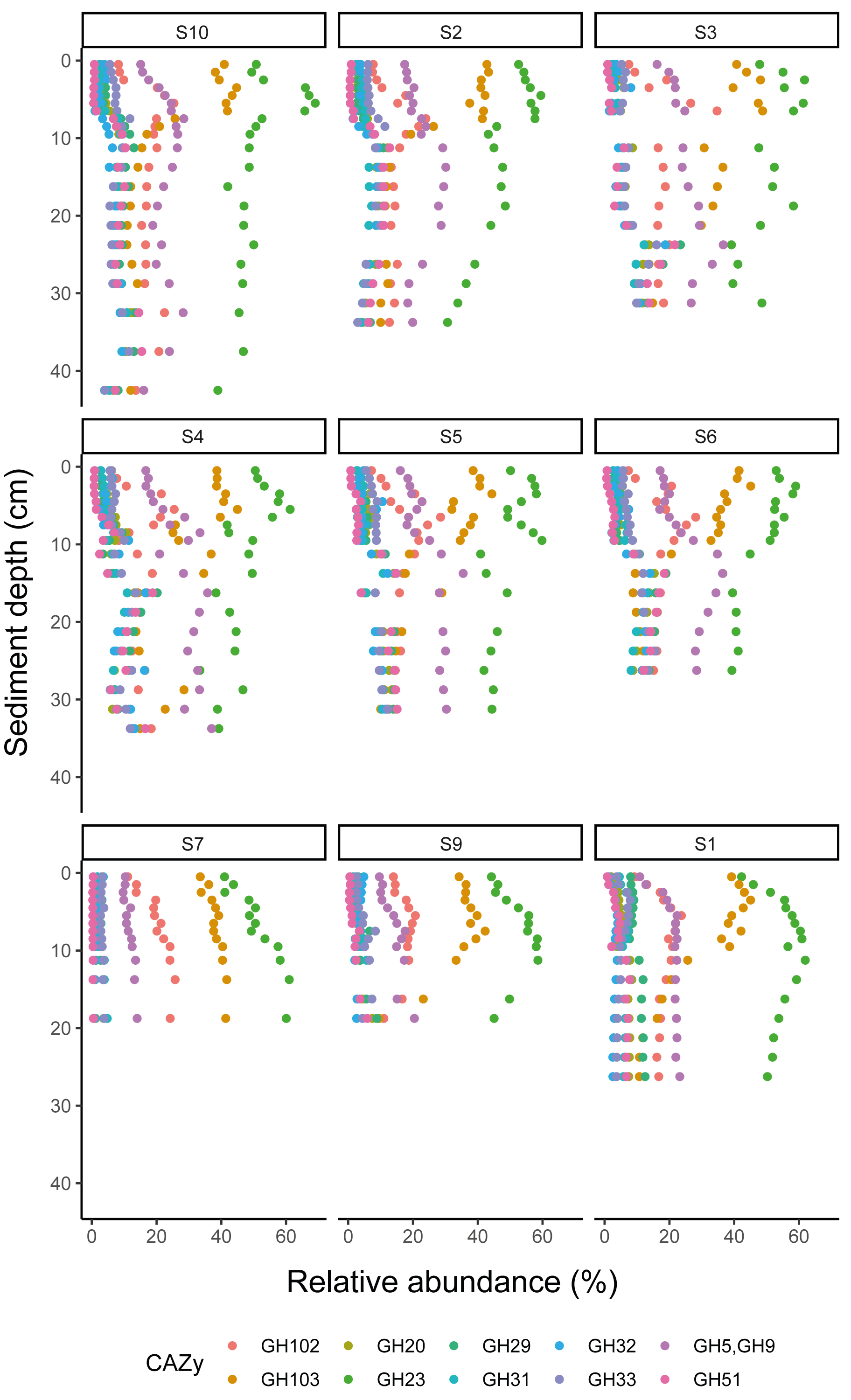


**Supplement Figure 7:** Distribution of carbohydrate-active enzyme (CAZyme) genes across sediment depth and geographic location. Each plot shows the relative abundances (x axis) of different CAZyme genes (represented by different colors) at nine different sites, plotted against sediment depth (y axis). The relative CAZyme abundance was estimated based on the relative abundance of the microbial genomes that encode them.


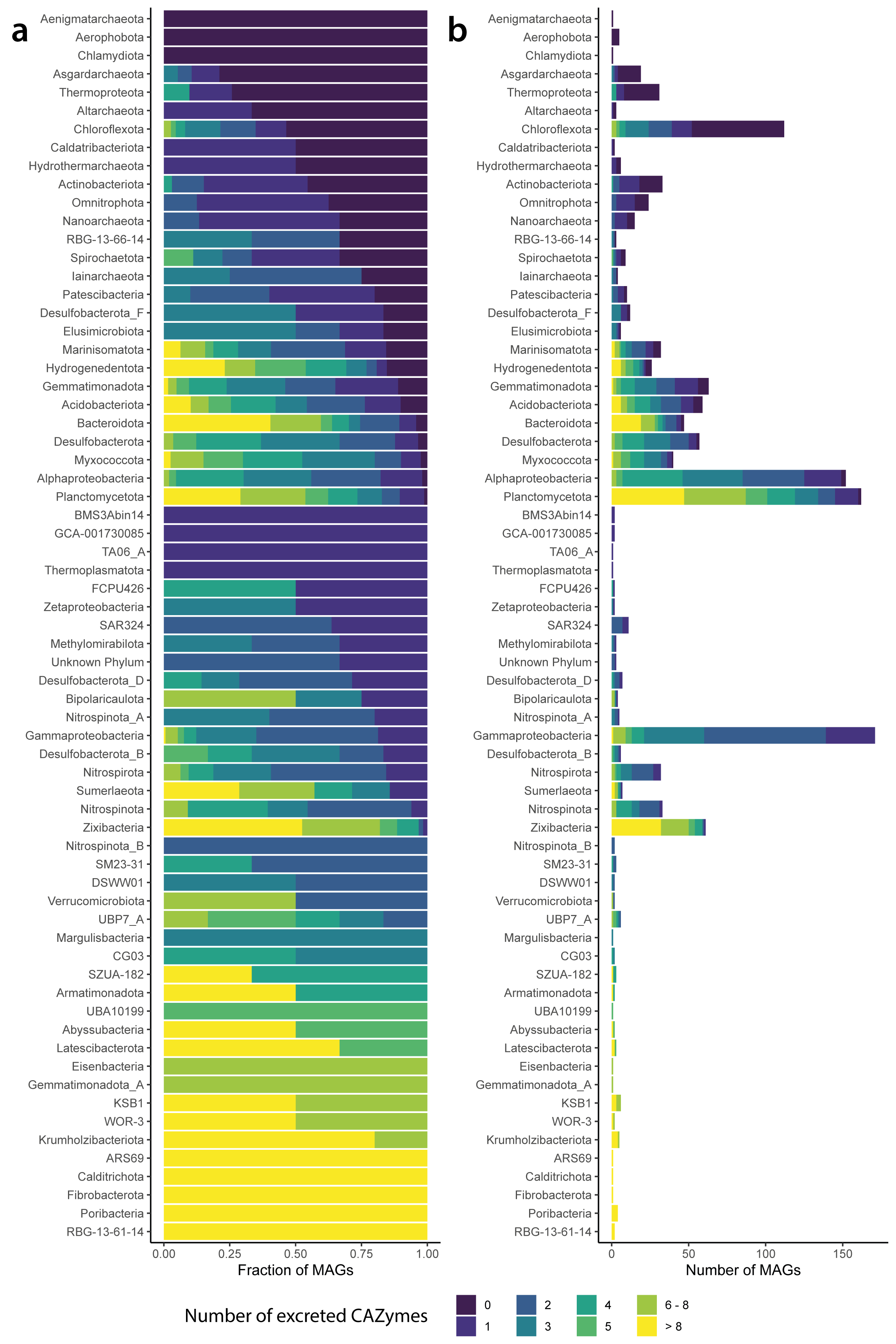


**Supplement Figure 8:** **a-b,** Classification of microbial genomes based on the number of carbohydrate-active enzymes (CAZymes) with signal peptides. The y-axis of both plots displays the microbial lineage, while the x-axis shows the relative fraction **(a)** or the number **(b)** of MAGs that contain 0, 1, 2, 3, 4, 5, 6-8, or more than 8 CAZymes genes with signal peptides for excretion within their genome. Each bar is colored based on the number of CAZymes within the microbial genomes.


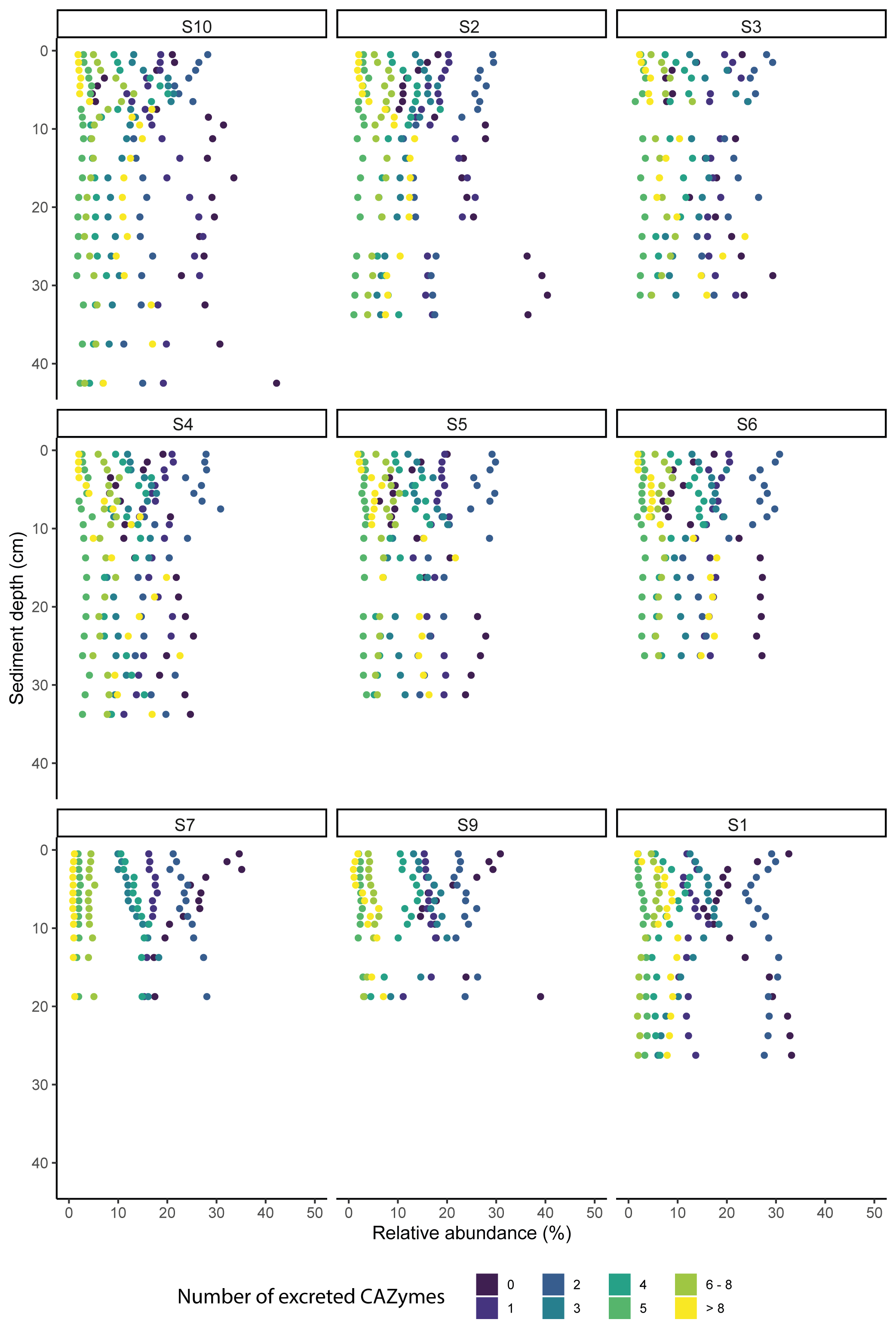


**Supplement Figure 9:** CAZyme diversity over sediment depth. Each plot shows the sum of relative abundances (x axis) of MAGs with different number of CAZymes within their genome (represented by different colors) at nine different sites, plotted against sediment depth (y axis).


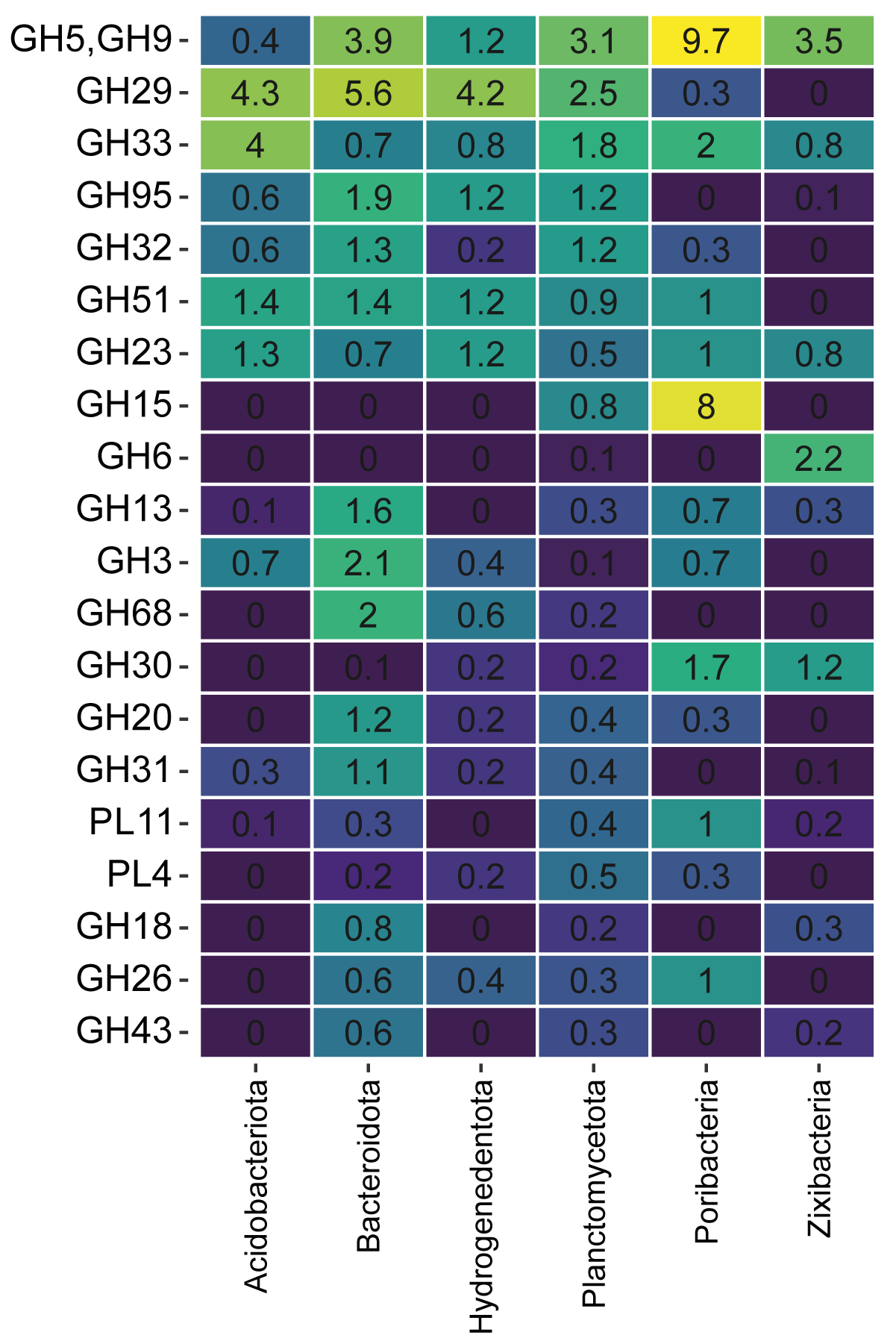


**Supplement Figure 10:** Average gene count of different CAZyme families and clusters within microbial lineages. The x axis shows the six microbial lineages where more than 3 MAGs had more than 8 different CAZymes with signal peptides for excretion in their genome. The y axis displays different CAZyme families / clusters, while the color gradient shows the average gene counts of CAZyme families / clusters within a microbial lineage.
